# Intra- and inter-individual metabolic profiling highlights carnitine and lysophosphatidylcholine pathways as key molecular defects in type 2 diabetes

**DOI:** 10.1038/s41598-019-45906-5

**Published:** 2019-07-04

**Authors:** Klev Diamanti, Marco Cavalli, Gang Pan, Maria J. Pereira, Chanchal Kumar, Stanko Skrtic, Manfred Grabherr, Ulf Risérus, Jan W. Eriksson, Jan Komorowski, Claes Wadelius

**Affiliations:** 1 Science for Life Laboratory, Department of Cell and Molecular Biology, Uppsala University, Uppsala, Sweden; 2 Science for Life Laboratory, Department of Immunology, Genetics and Pathology, Uppsala University, Uppsala, Sweden; 3 Department of Medical Sciences, Clinical Diabetes and Metabolism, Uppsala University, Uppsala, Sweden; 4 Translational Science & Experimental Medicine, Research and Early Development, Cardiovascular, Renal and Metabolism (CVRM), BioPharmaceuticals R&D, AstraZeneca, Gothenburg, Sweden; 5 Karolinska Institutet/AstraZeneca Integrated CardioMetabolic Center (KI/AZ ICMC), Department of Medicine, Novum, Huddinge, Sweden; 6 Pharmaceutical Technology & Development, AstraZeneca AB, Gothenburg, Sweden; 7 Department of Medicine, Sahlgrenska University Hospital, Gothenburg, Sweden; 8 Department of Medical Biochemistry and Microbiology, Uppsala University, Uppsala, Sweden; 9 Department of Public Health and Caring Sciences, Clinical Nutrition and Metabolism, Uppsala University, Uppsala, Sweden; 10 Institute of Computer Science, Polish Academy of Sciences, Warsaw, Poland

**Keywords:** Type 2 diabetes, Metabolomics

## Abstract

Type 2 diabetes (T2D) mellitus is a complex metabolic disease commonly caused by insulin resistance in several tissues. We performed a matched two-dimensional metabolic screening in tissue samples from 43 multi-organ donors. The intra-individual analysis was assessed across five key metabolic tissues (serum, visceral adipose tissue, liver, pancreatic islets and skeletal muscle), and the inter-individual across three different groups reflecting T2D progression. We identified 92 metabolites differing significantly between non-diabetes and T2D subjects. In diabetes cases, carnitines were significantly higher in liver, while lysophosphatidylcholines were significantly lower in muscle and serum. We tracked the primary tissue of origin for multiple metabolites whose alterations were reflected in serum. An investigation of three major stages spanning from controls, to pre-diabetes and to overt T2D indicated that a subset of lysophosphatidylcholines was significantly lower in the muscle of pre-diabetes subjects. Moreover, glycodeoxycholic acid was significantly higher in liver of pre-diabetes subjects while additional increase in T2D was insignificant. We confirmed many previously reported findings and substantially expanded on them with altered markers for early and overt T2D. Overall, the analysis of this unique dataset can increase the understanding of the metabolic interplay between organs in the development of T2D.

## Introduction

Type 2 diabetes (T2D) mellitus is a disease characterized by poor insulin sensitivity and failure of the pancreatic β-cells to secrete appropriate amounts of insulin^1,2^. Insulin resistance (IR) is associated with low physical activity, obesity, dyslipidemia and hypertension^3,4^. T2D is a major global health problem with a reported prevalence of 422 million in 2014 according to the World Health Organization (WHO)^5^. The insulin-related deficiencies in T2D have been associated with alterations in glucose, fatty acid uptake and metabolism, as well as lipid deposition in different tissues^6–8^.

The field of metabolomics has emerged through the development of high-throughput analytical chemistry techniques^2^. Metabolic profiling enables untargeted screening of a snapshot of the metabolic status for a given sample^9,10^. Liquid chromatography (LC) coupled with mass-spectrometry (LC-MS) and gas chromatography (GC) coupled with MS (GC-MS) are two prominent metabolic profiling technologies covering a wide spectrum of cellular low-weight compounds (<1500 Da)^9^. The untargeted screening is followed by computational “translation” of a subset of the identified compounds into known metabolites^11^.

IR in several tissues in combination with insufficient insulin secretion lead to systemic metabolic defects. Multiple studies have attempted to identify potential T2D biomarkers through metabolic profiling of blood, urine or saliva^1,9,10,12–15^. Biomarkers identified in biofluids may partly reflect ongoing metabolic processes throughout the body, but are secondary to events occurring in other tissues^16^. Adipose tissue, liver, pancreatic islets and skeletal muscle are well-known to contribute to T2D development^17^, but others like brain and gut are also of importance.

In this study we performed a metabolic screening in a cohort of 43 organ donors. The study spanned across five key metabolic tissues (blood serum, intra-abdominal/visceral adipose tissue (VAT), liver, pancreatic islets and skeletal muscle) for T2D. The subjects were selected from a cohort of more than 200 multi-organ donors from the EXODIAB biobank with no statistical difference for age, body-mass index (BMI) and gender across the different phenotypes to accurately capture the metabolic fingerprint of T2D onset. To our knowledge, this is the first attempt to create an intra-individual map of metabolites across various metabolic-relevant tissues for the same cohort of individuals. Our study confirms a large number of findings from the existing literature and substantially expands them with altered markers for early and overt T2D stages in analyzed tissues. In total we identified 286 unique metabolites across all tissues, 32% of which were significantly altered between non-diabetes and T2D. Amino acids (AAs) were elevated in VAT, liver, muscle and serum in T2D, and significantly associated to the percentage of glycosylated hemoglobin A_1c_ (HbA_1c_). Bile acids were similarly higher, but only in liver. We showed that carnitines and lysophosphatidylcholines (LPCs) are the most significantly altered metabolites in muscle, liver and serum in T2D. Finally, we explored the pair-wise differences among controls, pre-diabetes and overt T2D and suggested potential biomarkers especially for the pre-diabetes stage.

## Results

### Metabolic profiling

**Figure 1:**
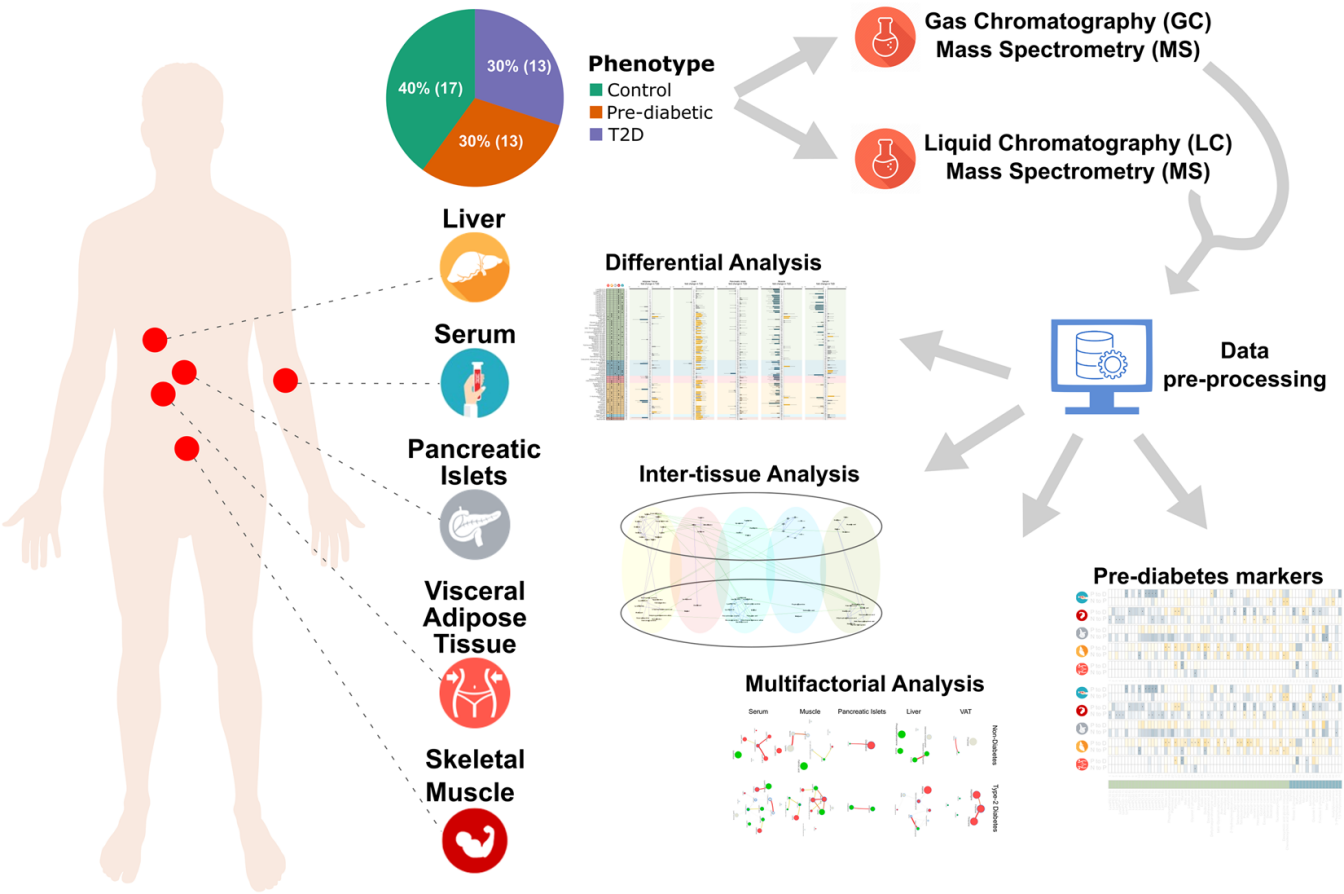
Schematic overview of the study summarizing sample collection, number of subjects, metabolic profiling, data processing and computational analysis.

**Figure 2:**
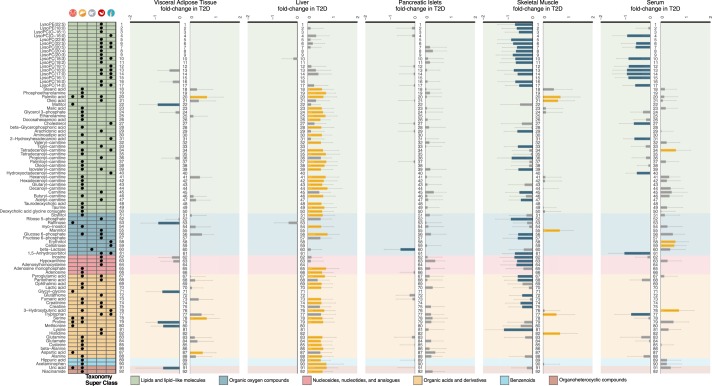
Overview of the differential and fold-change analysis of the computationally annotated compounds from GC-MS and LC-MS for non-diabetes versus T2D subjects. Rows of the table on the left-hand-side contain the 92 metabolites that were significant in at least one tissue. Table columns represent each of the five tissues (VAT, liver, pancreatic islets, skeletal muscle and serum). A black dot implies statistical significance in the corresponding tissue (Mann-Whitney U test permuted p-value < 0.1; Methods - Statistical analysis). The color-coding in the table originates from a curated classification of the HMDB super-class taxonomy and the labels are explained in the legend^51^. The five barplots represent the fold-change in T2D of the μ = 0 and σ = 1 scaled log_2_ compound intensities. Order of the barplots matches that of the tissues in the table. Error bars represent 90% confidence intervals (Methods - Statistical analysis). Yellow bars imply statistical significance and increase, blue bars statistical significance and decrease, while grey bars did not cross the statistical significance threshold. Numbering assists following the variation of metabolite across tissues.

We performed an untargeted metabolic profiling with GC-MS and LC-MS for 43 organ donors from healthy, pre-diabetes and T2D cohorts across five metabolic tissues: VAT, liver, pancreatic islets, skeletal muscle and blood serum (Fig. 1). The subjects were selected from a large collection of multi-organ donors from the EXODIAB biobank. There was no statistical difference between the groups for BMI, age and gender (Table 1; Supplementary Table S1). The putative identity of the metabolites was determined computationally and resulted in 142 and 144 unique metabolites for GC-MS and LC-MS, respectively. The samples were classified in three distinct phenotypes related to T2D (controls, pre-diabetes and T2D). Control and pre-diabetes subjects were merged into one non-diabetes group and compared to T2D (Table 1; Supplementary Fig. S1A,B). In total 92 unique metabolites varied significantly in at least one tissue between non-diabetes and T2D samples (Fig. 2).

**Table 1:** Baseline characteristics of the 43 subjects in the cohort.

Parameter	Controls	Pre-diabetes	Non-Diabetes	T2D	P-value 3C	P-value 2C
Gender	7 F/10 M	4 F/9 M	11 F/19 M	4 F/9 M	—	—
Age	59 ± 10	64 ± 8	61 ± 9	65 ± 7	0.2124	0.3013
BMI	26.5 ± 3.8	27.6 ± 5.6	27.0 ± 4.6	27.9 ± 5.6	0.8417	1
HbA_1c_	36.2 ± 1.9	40.8 ± 1.8	38.2 ± 2.9	56.5 ± 15.5	1.6 × 10^−8^	6.9 × 10^−7^
GSIS	11.8 ± 5.6	15.1 ± 30.8	13.2 ± 20.3	5.0 ± 2.5	1.3 × 10^−3^	2.6 × 10^−3^

The mean value and the standard deviation are shown for age, BMI, HbA_1c_ and GSIS. Gender is shown as the proportion of females (F) and males (M). Age is expressed in years. BMI is in kg/m^2^. HbA_1c_ is in % of mmol/mol. GSIS is mmol in liters of glucose. Non-diabetes is the merged group of controls and pre-diabetes. P-value 3C is calculated from a Kruskal-Wallis test on controls, pre-diabetes and T2D. P-value 2C is calculated from a Mann-Whitney U test on non-diabetes and T2D.

Intensities of internal standards (IS), deviation of experimental retention time and mass from their theoretical values and principal component analyses (PCAs) for the MS running order of samples were used to assess the quality of the profiling (Supplementary Note – Overview of metabolic profiling). IS varied mainly in LC-MS for VAT (Supplementary Figs S2 and S3). However, the relative standard deviation of the intensities in LC-MS was below 20% for all IS that ionize well in the respective mode. PCAs showed no evidence for effect of the running order (Supplementary Figs S4 and S5). Divergence of experimental retention time and mass for IS from the expected ones were within accepted limits (Supplementary Figs S6 and S7).

### Alterations of metabolites across tissues in T2D

After imputing and transforming the intensities of the metabolites we investigated the partitioning of the samples per tissue (Materials and methods – Data transformation and normalization). Sample grouping separation was ambiguous due to large within-group variation (Supplementary Fig. S8A). Covariates, excluding HbA_1c_ and glucose-stimulated insulin secretion (GSIS), were suggested (cf. Methods - Data transformation and normalization; Supplementary Table S2; Fig. S8) and proven (Supplementary Fig. S8B) to maintain the separation seen in the scaled and log_2_-transformed raw intensities (Supplementary Fig. S8A). Correcting for BMI, age, gender and sample weight did not yield extensive alterations in the set of significantly varying metabolites across tissues (Supplementary Fig. S9B).

Next, we sought to explore the statistical significance and the direction of the variation of the computationally annotated metabolite intensities between non-diabetes and T2D (Fig. 2). More than half of the total number of significant metabolites belonged to the taxon of lipids and lipid-like molecules, which mainly consisted of LPCs, carnitines and free fatty acids (NEFAs). Specifically, LPCs were overrepresented in muscle and serum of T2D (p-value < 10^−132^), while carnitines were abundant in liver of T2D (p-value < 10^−132^) (Supplementary Table S3; Fig. S10). We further observed that LPCs and carnitines in serum followed similar patterns with muscle and liver, respectively (Fig. 2). Various NEFAs were noticed as significant among liver, muscle and serum (Fig. 2).

Other important groups of metabolites were those of AAs and nucleosides, nucleotides and analogues. In T2D we additionally observed that glucose 6-phosphate levels were significantly higher in VAT and liver, while in muscle they were significantly lower, similarly to fructose 6-phosphate (Fig. 2). These metabolites, together with ribose 6-phosphate that is also significantly lower in muscle of T2D, are part of the pentose phosphate pathway that has been extensively linked to T2D^18^. We also observed a significant decrease of 1,5-anhydrosorbitol in both muscle and serum of T2D subjects. 1,5-Anhydrosorbitol, also known as 1,5-anhydroglucitol, is as an important short-term control factor for T2D in saliva and blood^19–22^.

**Figure 3:**
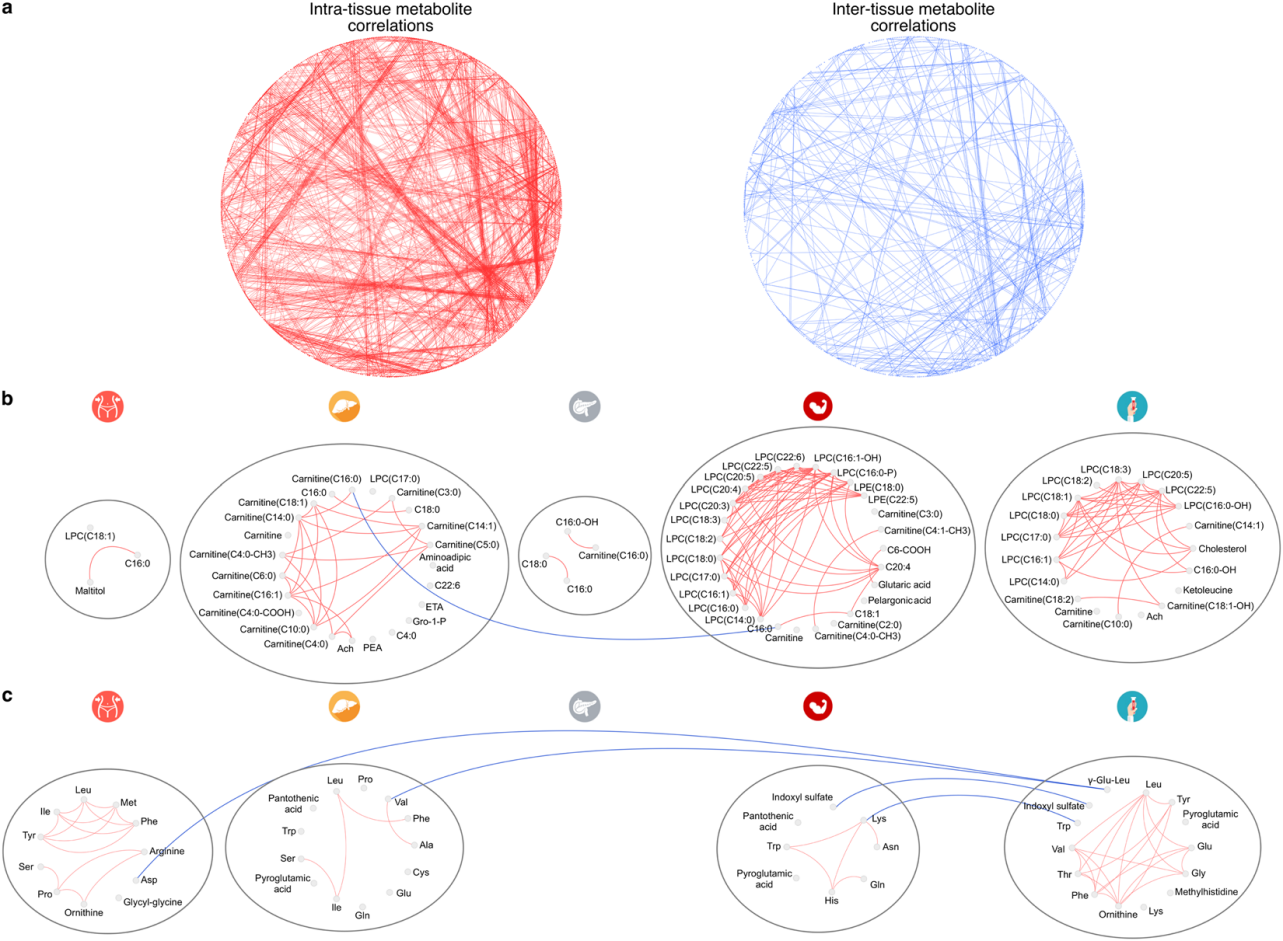
(**a**) MoDentify networks illustrating intra- (left panel) and inter-tissue (right panel) correlations of metabolites. Blue edges show metabolites correlated among tissues, while red, those correlated within tissues (p-value < 0.1) (Supplementary Note - MoDentify). (**b**,**c**) MoDentify networks for FAs (**b**) and AAs (**c**). Blue edges show metabolites correlated among tissues, while red, those correlated within tissues (p-value < 0.1). Nodes represent metabolites marked as significant in MoDentify (p-value < 0.1) (Supplementary Note - MoDentify), in T2D (Fig. 2; Methods – Statistical analysis) or in association with HbA_1c_ (Fig. 5; Methods – Statistical analysis). Each grey circle represents one tissue. Abbreviated metabolite names are explained in (Supplementary Table S4).

We also took advantage of the recent development of an R tool MoDentify^23^ to scan for metabolites correlated to the phenotype as single entities or as members of functional modules^24^. This analysis on the full set of metabolites revealed phenotype-driven functional modules, where single metabolites that are not directly correlated to T2D form combinatorial modules that are significantly associated to the phenotype (Supplementary Note - MoDentify). We confirmed that metabolites of the same taxon were strongly correlated^23^ (Supplementary Figs S11 and S12). Interestingly, we observed that the functional modules were mainly formed within tissues, rather than among tissues (Fig. 3A). The most enriched modules were those of metabolites belonging to the class of lipid and lipid-like molecules for liver, muscle and serum (Fig. 3B), which complements the prevalence of that class in the differential analysis (Fig. 2). LPCs in muscle and serum had high within group associations, while carnitines in liver were correlated to a lesser degree (Fig. 3B). Modules containing AAs, Branched-chain AAs (BCAAs) and aromatic AAs (AAAs) shared more inter-tissue connections, while VAT and serum had better intra-connected modules. In general, AAs showed a larger number of inter-tissue associations than the lipids (Fig. 3C).

### Regulation of carnitine and choline pathways

Carnitine represents a crucial *trait d’union* between carbohydrate and fatty acid (FA) metabolism. Carnitine facilitates the transport of FAs into the mitochondria for β-oxidation, but it is also involved in glucose metabolism regulating the pool of acetyl-CoA in the cytoplasm.

**Figure 4:**
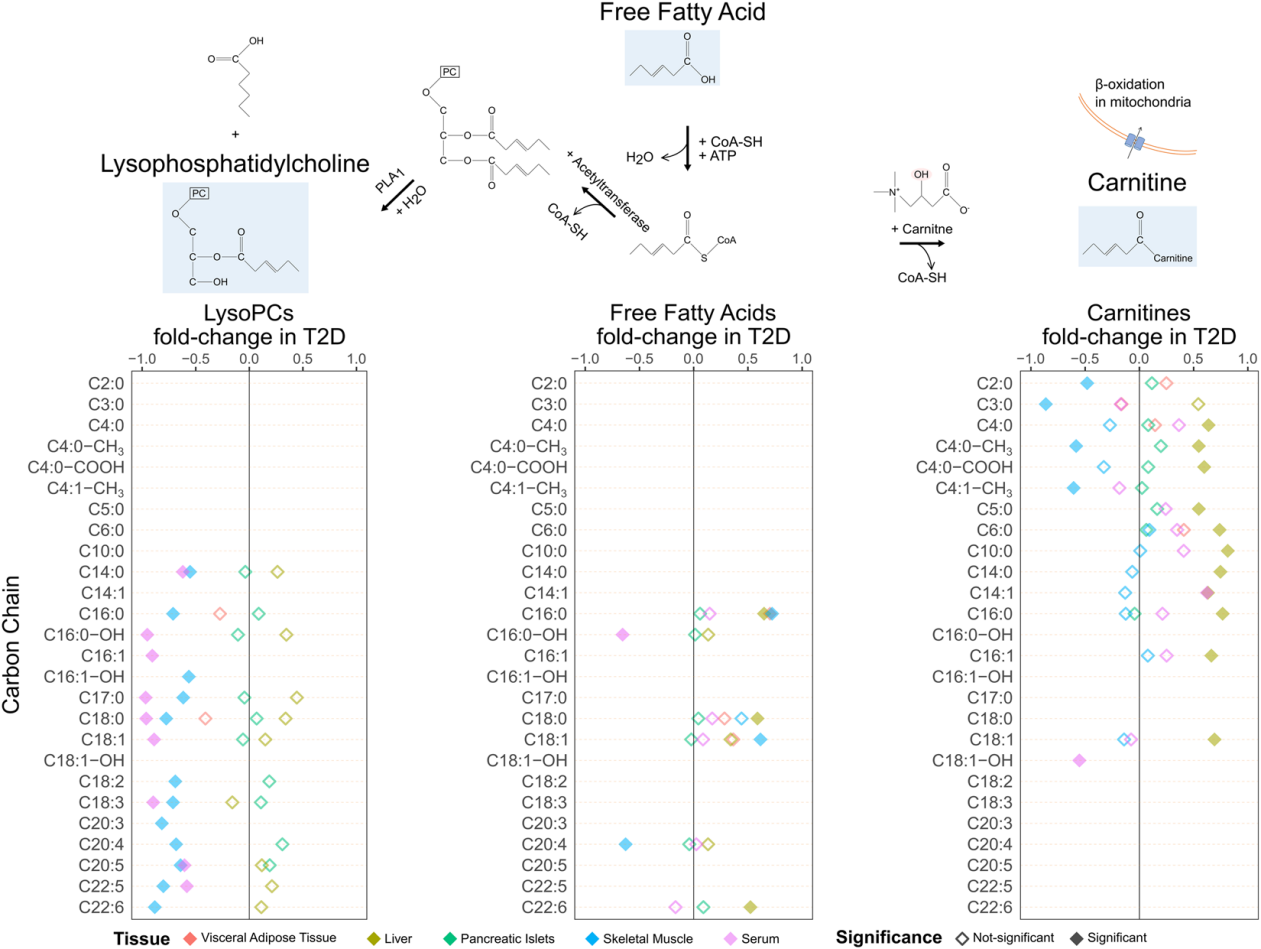
Schematic overview of the formation of LPCs and carnitines from NEFAs. The top graph shows an example of the pathway of LPCs and carnitines formation as conjugates of NEFAs (e.g. C6) with various other compounds via different enzymes. The three bottom plots describe the fold-change and significance of LPCs, NEFAs and carnitines (Mann-Whitney U test permuted p-value < 0.1; Methods - Statistical analysis). Rows represent carbon-chain length of the metabolites (Supplementary Table S4). Color-coding of the rhombuses represent tissues while the filling of the rhombus implies statistical significance.

In our samples, we observed a significant increase in the pool of short- and medium-chain carnitines in liver in T2D and a similar trend in pancreatic islets and serum. Instead, a trend towards reduction of carnitines was observed in muscle and in particular very short-chain carnitines were significantly reduced in T2D (Figs 2 and 4).

The complexity of the molecular mechanisms behind the association of carnitines to T2D was highlighted using a systems biology approach by Bene *et al*.^25^. They showed that individuals with type 1 diabetes or metabolic syndrome share similar levels of medium- and long-chain acyl-carnitines while individuals with T2D or metabolic syndrome have similar levels of short-chain acyl-carnitine.

We also found a generalized reduction in LPC levels in both serum and muscle including LPC with a surprisingly wide variety of saturation and desaturation; C14:0, C16:0, C16:1, C17:0, C18:0, C18:1, C18:2, C18:3, C20:4, C20:5, C22:5 and C22:6 (Fig. 4). The extent of decrease in LPCs is larger in muscle than in plasma (Fig. 2).

### Increased drive of bile acid pathway in T2D

We observed a statistically significant increase in the levels of deoxycholic acid conjugate with glycine or taurine in liver in T2D individuals, whereas the increase of chenodeoxycholic acid conjugates was not reaching statistical significance (Fig. 2). Glycodeoxycholate was also higher in liver, muscle and serum, but to a significant level only in liver. Taurine levels were also significantly higher in liver of T2D individuals with a non-statistically significant trend also in pancreatic islets, muscle and serum (Supplementary Fig. S13). Taurine, which has been reported to associate to serum glucose levels in animal models^26^, is synthesized in the pancreas from cysteine, an AA we detected as significantly elevated in T2D liver samples (Fig. 2; Supplementary Fig. S14). The pathways of primary bile acids biosynthesis and taurine-hypotaurine metabolism were significantly enriched in liver (p-value < 2.18 × 10^−2^) (Supplementary Table S5; Fig. S15).

### Amino acids vary across tissues in T2D

High levels of circulating BCAAs leucine, isoleucine, valine and the AAAs tryptophan, phenylalanine and tyrosine, have been repeatedly reported in the literature as associated to obesity, impaired fasting glucose and T2D^27^. Although the differences in BCAA and AAA did not cross the statistical significance threshold levels between control and T2D individuals, we observed a higher level of valine and isoleucine in serum in T2D (Supplementary Fig. S16). The same trend was observed in VAT. In liver and pancreatic islets the levels of valine, isoleucine and leucine were higher in T2D, while in muscle BCAAs were depleted in T2D (Supplementary Fig. S16). Tyrosine levels were elevated in VAT, muscle and serum and phenylalanine was higher in T2D liver, pancreatic islets and VAT samples (Supplementary Fig. S16). BCAAs, AAAs and other AAs that were significant either individually or as part of modules were shown to be strongly associated with T2D (Fig. 3C).

Adams and colleagues utilized plasma metabolite patterns to target muscle-specific metabolites in African-American females with significantly reduced whole-body lipid oxidation^12^. They found that higher levels of leucine and valine were strongly correlated with acetyl-carnitine, which we found significantly lower in muscle in T2D (Figs 2 and 4). Here we observed leucine, isoleucine and valine to be strongly correlated to acetyl-carnitine in muscle (Spearman correlation r > 0.65, p-value < 0.0102), which was also correlated to propionyl-carnitine (Spearman correlation r = 0.64, p-value < 0.0161), a marker of valine’s catabolic product propionyl-CoA^12,13^.

**Figure 5:**
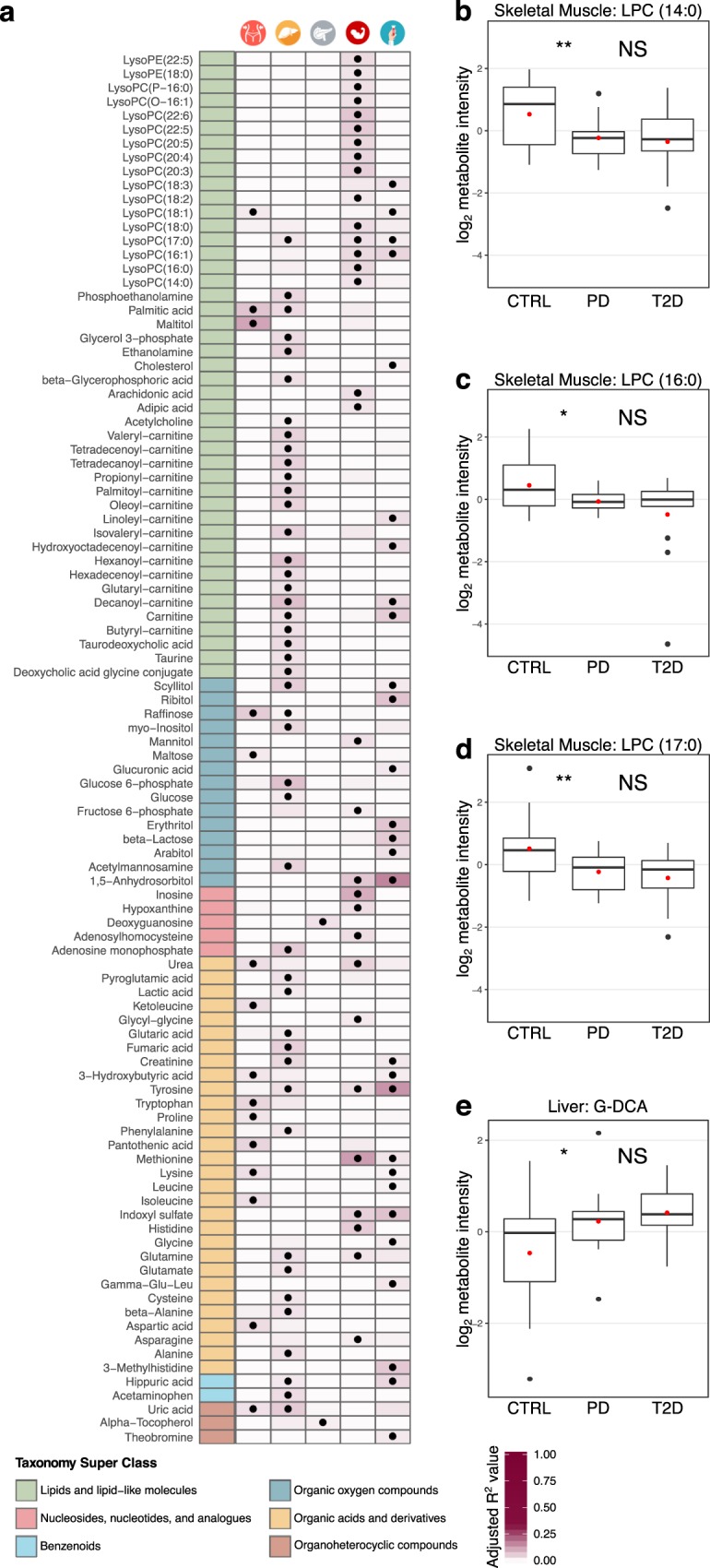
(**a**) Overview of the metabolites that are significantly linearly associated with HbA_1c_ in at least one tissue. The first column is color-coded according to a curated classification of the HMDB super-class taxonomy and the labels are explained in the legend. The following five columns represent each tissue as noted on the top of the heatmap. A black dot implies statistically significant association of the corresponding metabolite to HbA_1c_ (linear regression permuted p-value < 0.1; Methods – Statistical analysis). The color intensity in the cell background shows the level of the adjusted R^2^ value from the linear regression model. (**b**–**e**) Pair-wise comparisons of levels of selected metabolites between controls (CTRL) and pre-diabetes (PD), and pre-diabetes (PD) and T2D subjects. Statistical significance is shown as follows: NS, p-value > 0.1; *0.1 ≥ p-value > 0.05; **0.05 ≥ p-value > 0.01) (Mann-Whitney U permuted test; Methods – Statistical analysis). The red dot signifies the mean value of the group. (**b**) Pair-wise comparisons of LPC (14:0) in skeletal muscle; (**c**) Pair-wise comparisons of LPC (16:0) in skeletal muscle; (**d**) Pair-wise comparisons of LPC (17:0) in skeletal muscle; (**e**) Pair-wise comparisons of deoxycholic acid glycine conjugate (G-DCA) in liver.

Linear regression models showed that HbA_1c_ was associated to various BCAAs and AAAs for VAT, liver, muscle and serum. Specifically, we observed that tyrosine, phenylalanine, leucine and isoleucine in VAT were significantly associated with HbA_1c_ (Fig. 5A). Tyrosine and leucine shared the same significant pattern in VAT and serum (Fig. 5A; Supplementary Fig. S16). Interestingly tryptophan was significant for muscle and serum in T2D (Fig. 2), and significantly associated with HbA_1c_ in muscle, liver and serum, sharing an increasing pattern in liver and muscle, while decreasing in serum (Figs 2 and 5A; Supplementary Fig. S14). Elevated levels of tryptophan in serum is associated to T2D, making it a potential risk predictor for the pathogenesis of T2D^28^. There is increasing evidence pointing towards links between inflammatory processes and tryptophan-related metabolites, e.g. kynurenine that was higher in VAT, liver and muscle of T2D, but did not cross significance threshold (Supplementary Fig. S14)^29^.

Various other AAs have been associated to pre-diabetes or T2D with less consistency^30^. Particularly higher levels of alanine, serine, proline, glutamine, the glutamine/glutamate ratio, glutamate, histidine and lysine. Here we observed significantly higher levels of alanine, glutamine, glutamate, proline and serine in liver; significantly lower levels of proline and higher levels of serine in VAT; and significantly higher levels of histidine in muscle in T2D (Fig. 2; Supplementary Fig. S14). Higher levels of glutamate have been linked to diabetes due to its function as a substitute energy source to glycolysis or β-oxidation^2^.

Significantly lower levels of methionine and lysine were observed in T2D VAT and muscle, respectively (Fig. 2; Supplementary Fig. S14). Methionine and lysine are precursors involved in the endogenous synthesis of L-Carnitine which was also significantly lower in T2D muscle tissues (Fig. 2).

### FA-related pathways are enriched in T2D across tissues

Metabolite classification analysis revealed that VAT, liver, muscle and serum are enriched for AAs and various types of lipids. Specifically, VAT and serum were enriched for AAs (p-value < 1.15 × 10^−3^). Liver and muscle were enriched for AAs and derivatives (p-value < 1.13 × 10^−7^), and acyl carnitines (p-value < 2.3 × 10^−7^). Muscle was highly enriched for LPCs, similar to serum (p-value < 10^−132^) (Supplementary Table S3; Fig. S10). A pathway enrichment analysis showed various pathways related to lipids and lipid-like molecules, such as glycerophospholipid metabolism in liver (p-value < 4.63 × 10^−4^), biosynthesis of unsaturated FAs in muscle (p-value < 1.1 × 10^−3^) and carnitine synthesis in serum (p-value < 1.59 × 10^−2^) (Supplementary Table S5; Fig. S15). Overall, liver was the tissue with the largest number of significant metabolic pathways, followed by muscle, VAT and serum (Supplementary Fig. S17).

Liver, muscle and serum showed a strong enrichment in biological roles that are strongly connected to functionalities of lipids and lipid-like molecules in cells, such as lipid catabolism, fatty acid transport, energy production (p-value < 9.48 × 10^−5^). Muscle metabolites were enriched in unsaturated fatty acids (p-value < 1.99 × 10^−2^), and together with serum in membrane components and energy sources (p-value < 8.09 × 10^−2^) (Supplementary Table S6; Fig. S18). VAT, liver and muscle also showed enrichment for essential AAs (p-value < 4.03 × 10^−10^), and for various other AA-related biological roles (p-value < 1.08 × 10^−3^) (Supplementary Table S6; Fig. S18).

### Rule-based classification suggests combinations of metabolites that define type-2 deiabetes subjects

We performed a multivariate statistical analysis to verify metabolites identified by the earlier univariate analysis and to discover new metabolites that were non-linearly associated with T2D in various tissues. Indeed, a subset of LPCs was also marked as significant by the multivariate statistical approach in muscle and serum. In agreement with the previous analysis, proline was found significant in liver, while lysine and histidine in muscle. Proline was also marked as significant for muscle, even though the univariate approach did not mark it as such. Threonine and asparagine were found as significant in discerning between non-diabetes and T2D in VAT (Supplementary Fig. S19).

We next used the set of “all-relevant” selected metabolites from above as input to the ROSETTA machine-learning approach that produces transparent classification models to uncover combinations of variables that discriminate between phenotypes^31,32^. The computational models for liver, muscle and serum were of high quality (accuracy >80%), while those for VAT and pancreatic islets performed adequately well (Supplementary Table S7) given the complexity of the data and the sample size. Rough-set classification models consist of IF-THEN rules, which in addition to being legible, can be visualized into networks (Supplementary Table S8). Such networks show combinations of metabolites and their levels that differ between T2D and non-diabetes. In muscle combinations of high levels of palmitic acid and histidine, together with low levels of lysine and LPC (22:6) predict T2D, while average levels of LPC (22:6), mannitol and lysine predict non-diabetes (Supplementary Fig. S20). In liver various levels of adenosine monophosphate in combination with other AAs such as proline and alanine play a central role in deciding for T2D or non-diabetes (Supplementary Fig. S20). 1,5-Anhydrosobitol was selected as the strongest feature discriminating between the phenotypic classes and appeared to play a central role in the classification model for serum (Supplementary Fig. S19). Specifically, higher levels of 1,5-anhydrosobitol in combination with higher levels of specific LPCs predicted non-diabetes, while its moderate or low levels combination with medium or low levels of LPCs decided towards T2D (Supplementary Fig. S20).

### Variations in biomarker levels from controls to pre-diabetes and T2D subjects

In order to identify potential early markers of T2D we applied two different approaches. We used the group of pre-diabetes to monitor the development of metabolites through pair-wise comparisons of the significance between controls and pre-diabetes, as well as between pre-diabetes and T2D. We also expressed HbA_1c_, a marker of pre-diabetes, as a function of various metabolites via linear regression models (cf. Materials and methods).

We first explored the separation among the three phenotypes through PCA and observed that the separation was not satisfactory (Supplementary Fig. S2). This occurred mainly due to the limitations in statistical power and the large variation of the metabolite levels. The analysis of T2D and the group of pre-diabetes revealed that 45% of the significantly altered metabolites belonged to the class of lipids and lipid-like molecules (Supplementary Fig. S21A). A large proportion of these metabolites were significant when comparing pre-diabetes to T2D, but not when comparing controls and pre-diabetes. Liver showed a strong enrichment in significantly increased carnitines, NEFAs and various AAs in T2D, which were also associated with HbA_1c_ (Fig. 5A; Supplementary Fig. S21A). Compounds belonging to the class of nucleosides, nucleotides and analogues in muscle and six different LPCs in serum were significantly lower in T2D (Supplementary Fig. S21A). We also observed an extended association of various AAs, including several BCAAs and AAAs, with HbA_1c_ (Fig. 5A).

In contrast to serum, various LPCs and NEFAs in muscle showed a significant decrease in pre-diabetes when compared to controls, while the same compounds did not further alter or decrease in T2D (Fig. 5B–D; Supplementary Fig. S21A). Such a large collection of events of deregulation in pre-diabetes in combination with the large proportion of LPCs associated with HbA_1c_ suggested that specific LPCs in muscle might be early predictors for T2D (Fig. 5A–D). In serum and liver, it is also worth to observe that the levels of the bile acids pool increased between control and pre-diabetes while maintaining an elevated level in T2D subjects (Fig. 5E; Supplementary Fig. S21A). Changes in taurodeoxycholic acid, taurine and deoxycholic acid glycine conjugate were also significantly associated to the HbA_1c_ values in the liver (Fig. 5A). This provides additional evidence that imbalance of the cholesterol metabolism could represent an early marker of T2D.

## Discussion

We performed an extensive metabolomics analysis in a unique collection of five tissues of key importance for T2D. To our knowledge, this is the first attempt to investigate such a wide range of metabolic profiles across tissues in T2D. Our study provides a valuable dataset of metabolites for a large collection of tissues and our analysis contributes a detailed map of significant metabolites for T2D across these tissues. This map allowed us to confirm multiple known metabolites that serve as markers of T2D and to provide a fine-grained layer of additional metabolites and the tissue-of origin for a vast majority of them. Overall, metabolites formed within, rather than among group functional modules (Fig. 3A). In total 32% of all the detected metabolites were significant in at least one tissue when comparing non-diabetes to T2D. Generally, the levels of metabolites in liver and muscle, the two major insulin sensitive tissues, demonstrated divergent patterns, with metabolites in liver mainly increasing and the ones in muscle decreasing, potentially reflecting impaired tissue-specific IR and/or metabolic control (Fig. 2). We also observed a total increase of BCAAs and their associations with HbA_1c_, previously connected with IR in various tissues^27^ (Fig. 5A; Supplementary Figs S14 and S16). Reduced alanine levels in muscle and higher in liver in T2D, suggested an overall increase in the glucose-alanine cycle (Fig. 2; Supplementary Fig. S14).

Various AAs, including BCAAs and AAAs, were significantly associated with HbA_1c_ in VAT which clinically, is more interesting and relevant to T2D than subcutaneous adipose tissue (SAT). The VAT depot is overall more closely linked to IR and T2D phenotype, with potential causal effects on disturbing hepatic glucose metabolism in pre-diabetes^33^. Petrus *et al*. have earlier shown that adipose tissue depots differ in lipid and fatty acid composition, which may be due to distinct enzymatic activity, lipolytic function or gene expression^34^. Hence, our findings in VAT may not apply to other adipose depots such as SAT, but serum might be reflecting the module of VAT consisting of leucine, tyrosine and phenylalanine (Fig. 3C).

The largest class of metabolites that was altered in T2D samples was that of lipid and lipid-like molecules, with LPCs and carnitines highly enriched, and sharing patterns seen in serum with muscle and liver, respectively (Figs 2 and 4). Plasma LPC levels are determined by lecithin-cholesterol acyltransferase (LCAT) activity, hepatic secretion and phospholipase A2 synthesis from PC^35^. Reduction of LPCs in plasma has been reported to be tightly associated with obesity^35–37^ and IR^37–40^, while their elevated levels have been associated with glucose-lowering and anti-inflammatory effects^41^. A recent large study in humans found strong associations between LPC levels and insulin sensitivity in muscle, while the latter did not apply to insulin sensitivity in liver, suggesting a muscle-specific interplay between LPCs and insulin sensitivity^42^. Our observations of systematic decrease of LPCs in muscle and plasma, but not in liver, when comparing the T2D and control subjects, extends these findings and may be a strong indicator of diabetes (Figs 2 and 4). Pair-wise comparisons of controls to pre-diabetes and pre-diabetes to T2D showed a decreasing trend of LPCs in muscle. Specifically, LPCs were significantly decreased in muscle of pre-diabetes and while we observed a further decrease in T2D, it did not cross the threshold of statistical significance (Fig. 5B–D; Supplementary Fig. S21A). A study by Bruce and colleagues examined the impact of high-fat diet in LPCs of mice and detected similarly lower levels in the first week, indicating potential acute alterations^36^. Our results extend such indications to human subjects and provide a collection of LPC to be prioritized for further investigations.

The endogenous carnitine pool is for the major part located in skeletal muscle. Carnitines and acyl-carnitines are also present in the gastrointestinal tract, the liver and the kidneys. Metabolic changes can affect the carnitine pools in the various tissues, but the homeostasis can vary between tissues so that for example, changes in carnitine content in liver rapidly appear in plasma, whereas changes in skeletal muscle content may not be as readily detected^43^. Most of the metabolic studies of the correlation between carnitines, IR and T2D so far relied on analyses of urine, serum and plasma. Here we extended the analysis of the carnitine pool in T2D in other relevant tissues such as liver, muscle, VAT and pancreatic islets (Figs 2, 4 and 5A).

Alterations in the levels of long and medium chain acyl-carnitines, including octadecenoyl-carnitine (C18:1), tetradecenoyl-carnitine (C14:1), tetradecadienoyl-carnitine (C14:2) and short chain acyl-carnitines, such as malonyl-carnitine/hydroxybutyryl-carnitine (C3DC + C4OH), have been reported both in T2D and pre-diabetes states in serum^3,13,44,45^. Here we observed a general increase in the short and medium-chain carnitines in liver in T2D, and a similar pattern in serum and pancreatic islets (Figs 2, 4 and 5A; Supplementary Fig. S21A). Short and medium-chain carnitines in liver also formed a strongly interconnected module that highlights the importance of the group in T2D (Fig. 3B). Accumulation of long-chain acyl-carnitines and acyl-carnitine-species in T2D plasma might reflect an increased, yet incomplete, mitochondrial β-oxidation with even-chain acyl-carnitines, up to 20 carbons, resulting from the initial rounds of oxidation and odd-chain species associated to AA catabolism. The deregulation of β-oxidation seems to be unrelated to the activity of the carnitine acyltransferase I (CPT1) enzyme^46^. Additionally, 3-hydroxybutyric acid, a member of the ketone bodies, whose increase is known to be associated with enhanced β-oxidation, was higher in muscle and significantly higher in liver and serum (Fig. 2)^47,48^.

Bile acids have been associated with the regulation of cholesterol catabolism, lipid-absorption, homeostasis of triglycerides and glucose. They can also act as hormones regulating various metabolic processes. T2D has been linked to variation of the bile acids pool composition, in particular the ratio of the pool of cholic and deoxycholic acid in the liver to chenodeoxycholic acid in the intestine is reported to be a metabolic signature of impaired cholesterol catabolism in T2D. Evidence in animal models and human points to a higher level of cholic acid synthesis in T2D, which in turn increases the level of deoxycholic acid^4,49,50^. Here we observed higher levels of bile acids in liver, muscle and serum of T2D (Fig. 2; Supplementary Fig. S13). A closer look at the levels of bile acids revealed strong correlations to HbA_1c_ and increased levels in liver and serum in pre-diabetes and T2D (Figs 5A,E; Supplementary Fig. S21A).

Carbohydrate metabolites such as glucose- and fructose-6-phosphate are increased in VAT, liver and serum, but reduced in muscle in T2D. This might reflect reduced metabolism in muscle due to IR and increased supply to other organs (Fig. 2). 1,5-Anhydrosorbitol is known to be decreased in blood and saliva of T2D subjects^19–22^. In our study, in addition to serum, we also found it to be significantly lower in the muscle of T2D, which we believe is the primary event and is reflected in biofluids. Decanoyl-carnitine and deoxycholic acid glycine conjugate show the same significantly increased pattern in serum and liver of pre-diabetes (Supplementary Fig. S21A). These serum markers might reflect their corresponding levels in liver as potential early markers for IR.

Despite the novel findings on metabolite changes across several tissues, this study has clear limitations. Due to the stringent ethical regulations of the Swedish human transplantation organization, we did not have access to all confounding factors. Hence, there are likely effects due to the acute disease conditions as well as medications and other interventions at the intensive care unit, including metabolic effects of stress. The results need to be confirmed in other settings, e.g. in cohorts of deceased donors with a larger set of potentially confounding factors. However, large international projects such as the Genotype-Tissue Expression (GTEx), have extensively utilized biobanks that enable extensive exploratory analyses in multiple tissues that are challenging to obtain from living donors. Additionally, we confirmed a large number of findings from the available literature that support our approach. There was also lack of statistical power due to the limited collection of samples and, moreover, causality could not be inferred. Instead we explored sets of potential biomarkers that differed between pre-diabetes or T2D. Increasing the number of samples would partly overcome this limitation, and allow future investigations of the dysregulated molecular mechanisms. Further limitations are due to the complex extraction of metabolites from VAT, the relative compound quantification from MS and the uncertainty of the computational pairing of peaks to compounds. However, these limitations are expected to be addressed together with the constant advancements in the field of MS and standardization of complex sample preparation protocols.

## Materials and Methods

### Ethics statement

The use of human organs for transplantation and scientific research is regulated by the Swedish law. A collaborative initiative between the universities of Uppsala and Lund created the Human Tissue Laboratory (HTL) funded by a strategic grant from the Excellence of Diabetes Research in Sweden (EXODIAB). They have created a large biobank of tissue samples from deceased human multi-organ donors. Tissue samples (n = 43) for VAT, skeletal muscle, liver, pancreatic islets and blood serum were obtained through the EXODIAB network following the Uppsala Regional Ethics Committee approval (Dnr: 2014/391). Informed consent to use the organs for scientific research was obtained from the donors or their legal guardians. The samples were stored and analyzed in full accordance with the regional standard practices and the Swedish law. No organs/tissues were procured from prisoners.

### Sample collection and processing

We randomly selected subjects with normoglycemia (n = 17), with pre-diabetes (n = 13) and with T2D (n = 13) out of the EXODIAB multi-organ organ donor biobank (n > 200) (Fig. 1; Table 1; Supplementary Table S1). Samples were acquired from frozen portions of five metabolically-relevant human tissues (VAT, liver, pancreatic islets, abdominal skeletal muscle and blood serum). T2D patients were identified based on their medical records at the corresponding medical facilities according to the WHO guidelines^5^. We defined pre-diabetes subjects by the percentage of HbA_1c_ level (5.7 ≤ HbA_1c_ ≤ 6.5%) in blood (Table 1). We classified samples with HbA_1c_ < 5.7 as controls (Table 1). We merged control and pre-diabetes subjects into one group (non-diabetes) into a primary analysis, and compared their metabolic profiling to the one of T2D. We also measured the GSIS in pancreatic islets that proved to be correlated to be impaired in T2D patients^51^ (Table 1; Supplementary Table S1; Fig. S22). In secondary analyses, we also examined all three groups separately.

### Metabolic profiling

Metabolic profiling with GC-MS and LC-MS was performed at the Swedish Metabolomics Center in Umeå, Sweden. Detailed information about sample preparation, mass spectrometry and targeted data processing is available at (Supplementary Note - Metabolic Profiling). The remaining supernatants of each tissue type were pooled and used to create tissue-specific quality control (QC) samples. Tandem mass spectrometry analysis (LC-MS only) was run on the QC samples for identification purposes. The samples were analyzed in per-tissue batches according to a randomized run-order on both GC-MS and LC-MS.

### Data transformation and normalization

Metabolites missing in more that 25% of the samples were removed. log_2_ transformation was applied to the whole dataset after missing values were replaced by 1.00001 to avoid negative values. The transformation assisted in achieving a better approximation of a normal distribution and avoiding bias by outliers^7^. Quantifications from GC-MS and LC-MS were merged into one dataset and metabolite intensities were scaled to μ = 0 and σ = 1^52^.

ISs from MS were also included to the set of covariates, while HbA_1c_ and GSIS were excluded due to their associations with the outcome (Table 1; Supplementary Table S1; Fig. S22). Assessment for the contribution of covariates to the separation of samples into groups was performed by applying a generalized linear model that employs a maximum likelihood optimization approach (glmnet)^53^. Glmnet combines the advantages of least absolute shrinkage and selection operator (lasso) that tends to select the most correlated covariate and ridge penalty that tends to shrink the coefficients of the correlated covariates, and take advantage of their potentially linear combinatorics. We ran glmnet with lasso and ridge penalty contributing equally to the model (α = 0.5) on a 10-fold cross validation mode in order to achieve an optimal set of lambda coefficients. The coefficients proved to be unimportant (Supplementary Table S2; Figs S1B and S8B). We further investigated whether the detected metabolites were highly associated to covariates by performing Spearman correlation tests while considering the corresponding false discovery rate (FDR) adjusted p-values (Supplementary Table S9). Finally, we explored the structure of the sets of samples with tissue-specific principal component analyses (PCAs) (Supplementary Figs S1 and S8).

### Statistical analysis

We applied non-parametric (Mann Whitney U test) permutation tests to estimate the statistical importance of the intensities of the metabolites. We used the R package “coin” that employs Monte Carlo resampling to build the background distribution of the test statistic for a given dataset. We performed 100 K permutations and set the threshold for statistical significance to 0.1. We additionally computed the fold-change and 90% confidence intervals of the average intensities of metabolites for binary phenotypic groups^9^, non-diabetes and T2D.

We applied a similar approach to explore the non-monotonic levels of metabolites for the pair-wise phenotypic groups of controls, pre-diabetes and T2D. Specifically, we focused on the comparison of controls to pre-diabetes, and pre-diabetes to T2D. For the same purpose we also explored linear regression models of HbA_1c_ expressed as a function of various single metabolites and measured their estimation of the proportion of variance explained by the computed regression equation (R^2^) and their p-values. For the linear regression models we used the R package lmPerm that uses permutation tests (100 K permutations) to build the background distribution for the estimation of statistical significance.

### Multivariate analysis and classification

Additionally, we performed a multivariate variable selection analysis using Boruta^31^. Boruta tackles the problem of selecting *all-relevant* variables that best discern between the phenotypic classes employing random forest classifiers and comparing the relevance of real variables to random synthetic probes. Next we used the selected set of significant metabolites to build tissue-specific classifiers using an R wrapper for the rough-set ROSETTA toolkit^32,54^. ROSETTA computes classifiers in the form of minimal IF-THEN rules that are used to predict an *a priori* defined decision, which in this case was non-diabetes or T2D. In the next step the rules were explored in a network format called VisuNet that facilitates visual exploration of the classifier and demonstrates the co-predictive power of combinations of metabolites^55^.

### Computational analysis tools

To maximize the available information for each metabolite we merged two of the current most widely used small molecules databases, the human metabolome database (HMDB)^56^ and the chemical entities of biological interest (ChEBI)^57^. We also included information from two metabolic-pathway databases, the Kyoto Encyclopedia of Genes and Genomes (KEGG)^58^ and the small molecule pathway database (SMPDB)^59^. Such an integrated database helped us retrieve various information from multiple existing databases for computationally annotated metabolites detected by high-throughput metabolomics technologies (Supplementary Table S10). We implemented a computational tool that converted the information from various metabolomics databases to a unified local collection of data. The tool is available under https://github.com/klevdiamanti/metabolomicsDB.

We additionally implemented a computational tool that performed an integrated analysis of the metabolite intensities, and it is freely available under https://github.com/klevdiamanti/MS_targeted. It takes as input a collection of pre-processed metabolite measurements and their identifiers, and it outputs a set of analysis results that include: (i) differential statistical analysis; (ii) visual exploration of the statistical analysis; (iii) correlation of metabolites and covariates; (iv) visual exploration of correlations of metabolites and covariates; (vi) datasets for machine-learning approaches; (v) correlation analysis for pairs of metabolites; (vi) overrepresentation of compounds in metabolic pathways; (vii) input datasets for MoDentify^23^; (viii) ratio of concentrations for metabolite pairs^60^.

## Supplementary information

Supplementary Material

Dataset 1

## Data Availability

The metabolomics datasets generated and analyzed during the current study are available in MetaboLights (https://www.ebi.ac.uk/metabolights/) under the accession number MTBLS690.
