# Author Correction: Intra- and inter-individual metabolic profiling highlights carnitine and lysophosphatidylcholine pathways as key molecular defects in type 2 diabetes

**DOI:** 10.1038/s41598-019-54423-4

**Published:** 2019-11-28

**Authors:** Klev Diamanti, Marco Cavalli, Gang Pan, Maria J. Pereira, Chanchal Kumar, Stanko Skrtic, Manfred Grabherr, Ulf Risérus, Jan W. Eriksson, Jan Komorowski, Claes Wadelius

**Affiliations:** 1 Science for Life Laboratory, Department of Cell and Molecular Biology, Uppsala University, Uppsala, Sweden; 2 Science for Life Laboratory, Department of Immunology, Genetics and Pathology, Uppsala University, Uppsala, Sweden; 3 Department of Medical Sciences, Clinical Diabetes and Metabolism, Uppsala University, Uppsala, Sweden; 4 Translational Science & Experimental Medicine, Research and Early Development, Cardiovascular, Renal and Metabolism (CVRM), BioPharmaceuticals R&D, AstraZeneca, Gothenburg, Sweden; 5 Karolinska Institutet/AstraZeneca Integrated CardioMetabolic Center (KI/AZ IC MC), Department of Medicine, Novum, Huddinge, Sweden; 6 Pharmaceutical Technology & Development, AstraZeneca AB, Gothenburg, Sweden; 7 Department of Medicine, Sahlgrenska University Hospital, Gothenburg, Sweden; 8 Department of Medical Biochemistry and Microbiology, Uppsala University, Uppsala, Sweden; 9 Department of Public Health and Caring Sciences, Clinical Nutrition and Metabolism, Uppsala University, Uppsala, Sweden; 10 Institute of Computer Science, Polish Academy of Sciences, Warsaw, Poland

Correction to: *Scientific Reports* 10.1038/s41598-019-45906-5, published online 04 July 2019

The original version of this Article contained an error in Affiliation 4, which was incorrectly given as ‘Translational Science & Experimental Medicine, Early Cardiovascular, Renal and Metabolism, R&D BioPharmaceuticals, AstraZeneca, Gothenburg, Sweden’. The correct affiliation is listed below:

Translational Science & Experimental Medicine, Research and Early Development, Cardiovascular, Renal and Metabolism (CVRM), BioPharmaceuticals R&D, AstraZeneca, Gothenburg, Sweden

This error has now been corrected in the HTML and PDF versions of the Article as well as the Supplementary Information.

