# Supplementary material for: Intra- and inter-individual metabolic profiling highlights carnitine and lysophosphatidylcholine pathways as key molecular defects in type 2 diabetes
Source: Sci Rep. 2019 Jul 4;9:9653. doi: 10.1038/s41598-019-45906-5 (PMC6609645; doi:10.1038/s41598-019-45906-5)
Supplement: Supplementary file 1 — Supplementary Material [file 41598_2019_45906_MOESM1_ESM.pdf]

Intra- and inter-individual metabolic profiling highlights carnitine and lysophosphatidylcholine pathways as key molecular defects in type 2 diabetes

Klev Diamanti[1], Marco Cavalli[2], Gang Pan[2], Maria J Pereira[3], Chanchal Kumar[4,5], Stanko Skrtic[6,7], Manfred Grabherr[8], Ulf Risérus[9], Jan W Eriksson[3], Jan Komorowski[1,10] and Claes Wadelius[2,*]

[1]Science for Life Laboratory, Department of Cell and Molecular Biology, Uppsala University, Sweden
[2]Science for Life Laboratory, Department of Immunology, Genetics and Pathology, Uppsala University, Sweden
[3]Department of Medical Sciences, Clinical Diabetes and Metabolism, Uppsala University, Uppsala, Sweden
[4]Translational Science & Experimental Medicine, Research and Early Development, Cardiovascular, Renal and Metabolism (CVRM), BioPharmaceuticals R&D, AstraZeneca, Gothenburg, Sweden
[5]Karolinska Institutet/AstraZeneca Integrated CardioMetabolic Center (KI/AZ ICMC), Department of Medicine, Novum, Huddinge, Sweden
[6]Pharmaceutical Technology & Development, AstraZeneca AB, Gothenburg, Sweden
[7]Department of Medicine, Sahlgrenska University Hospital, Gothenburg, Sweden
[8]Department of Medical Biochemistry and Microbiology, Uppsala University, Sweden
[9]Department of Public Health and Caring Sciences, Clinical Nutrition and Metabolism, Uppsala University, Sweden
[10]Institute of Computer Science, Polish Academy of Sciences, Poland
[*]Corresponding author

Supplementary Material

SUPPLEMENTARY NOTE

METABOLIC PROFILING

We performed an untargeted metabolic profiling with gas chromatography (GC) mass spectrometry (MS) and liquid chromatography[1] MS for 43 organ donors across five metabolic tissues (intra-abdominal/visceral adipose tissue (VAT), liver, pancreatic islets, skeletal muscle and blood serum)[1–4].

SAMPLE PREPARATION

INTRA-ABDOMINAL/VISCERAL ADIPOSE TISSUE

500µL of 2/1(v/v) CHCl3:methanol (including D4-Cholic Acid) and, 100µL water (including 13C9-phenylalanine) and 2 tungsten beads were added to each sample (18-23mg) (Supplementary Note - Metabolic Profiling). The samples were shaken at 30Hz for 3 minutes. The tungsten beads were removed, and the samples were left standing at room temperature for 30 minutes. The samples were centrifuged at 14000rpm, +4°C for 3 minutes and 80µL of the aqueous phase were transferred to Eppendorf tubes. 320µL methanol (including D6-salicylic acid) was added to the Eppendorf tubes, whereupon remaining proteins were precipitated at -20°C for 1 hour. The samples were centrifuged for 10 minutes at 14000rpm, +4°C and 50µL supernatant were taken out to GC vials and 200µL for LC-MS. Solvents were evaporated and the samples were stored at -80°C until analysis.

LIVER, MUSCLE AND PANCREATIC ISLETS

1000µL (450µL for islets) of extraction buffer (80/20 v/v methanol: water) including internal standards (Supplementary Note - Metabolic Profiling) were added to 9-12mg of tissue. The sample was shaken with a tungsten bead in a mixer mill at 30Hz for 3 minutes, the bead was removed and the sample was centrifuged at +4°C, 14000rpm, for 10 minutes. The supernatant was transferred to micro vials and evaporated to dryness in a speed-vac concentrator. For liver and muscle tissues 200µL was used for LCMS and 200µL for GCMS analysis. For islets, 200µL of the supernatant was evaporated for LCMS and 100µL for GCMS analysis[5,6].

SERUM

900 µL of extraction buffer (90/10 v/v methanol: water) including internal standards for both GC-MS and LC-MS (Supplementary Note - Metabolic Profiling) were added to 100µL of serum. The sample was shaken at 30Hz for 2 minutes in a mixer mill and proteins were precipitated at +4°C on ice. The sample was centrifuged at +4°C, 14000rpm, for 10 minutes. The supernatant, 200µL for LC-MS analysis and 200µL to GC-MS analysis, was transferred to micro vials and evaporated to dryness in a speed-vac concentrator[5].

MASS SPECTROMETRY

LC-MS

Before the LC-MS analysis the sample was re-suspended in 10+10µL methanol and water. Each batch of samples was first analyzed in positive mode. After all samples within a batch were analyzed, the instrument was switched to negative mode and a second injection of each sample was performed.

The chromatographic separation was performed on an Agilent 1290 Infinity UHPLC-system (Agilent Technologies, Waldbronn, Germany). 2µL of each sample were injected onto an Acquity UPLC HSS T3, 2.1×50mm, 1.8µm C18 column in combination with a 2.1×5mm, 1.8µm VanGuard precolumn (Waters Corporation, Milford, MA, USA) held at 40°C. The gradient elution buffers were A (H2O, 0.1% formic acid) and B (75/25 acetonitrile:2-propanol, 0.1% formic acid), and the flow-rate was 0.5mL/min. The compounds were eluted with a linear gradient consisting of 0.1-10% B over 2 minutes, B was increased to 99% over 5 minutes and held at 99% for 2 minutes; B was decreased to 0.1% for 0.3 minutes and the flow-rate was increased to 0.8mL/min for 0.5 minutes; these conditions were held for 0.9 minutes, after which the flow-rate was reduced to 0.5mL/min for 0.1 minutes before the next injection.

The compounds were detected with an Agilent 6550 Q-TOF mass spectrometer equipped with a jet stream electrospray ion source operating in positive or negative ion mode. The settings were kept identical between the two modes, with the difference lying in the capillary voltage. A reference interface was connected for accurate mass measurements; the reference ions purine (4µM) and HP-0921 (Hexakis(1H, 1H, 3H-tetrafluoropropoxy)phosphazine) (1µM) were infused directly into the MS at a flow rate of 0.05 mL/min for internal calibration, and the monitored ions were purine m/z 121.05 and m/z 119.03632; HP-0921 m/z 922.0098 and m/z 966.000725 for positive and negative mode respectively. The gas temperature was set to 150°C, the drying gas flow to 16L/min and the nebulizer pressure 35psig. The sheath gas temperature was set to 350°C and the sheath gas flow 11L/min. The capillary voltage was set to 4000V in positive ion mode, and to 4000V in negative ion mode. The nozzle voltage was 300V. The fragmentor voltage was 380V, the skimmer 45V and the OCT 1 RF Vpp 750V. The collision energy was set to 0V. The m/z range was 70-1700, and data was collected in centroid mode with an acquisition rate of 4 scans s-1 (1977 transients/spectrum).

GC-MS

Derivatization and GC-MS analyses were performed as described previously[5]. The pancreatic islets and the VAT were derivatized in a final volume of 30µL, rather than 90µL, which was used for all the other tissues. 1µL of the derivatized sample was injected in splitless mode by a CTC Combi Pal autosampler (CTC Analytics AG, Switzerland) into an Agilent 6890 gas chromatograph equipped with a 10m×0.18mm fused silica capillary column with a chemically bonded 0.18µm DB 5-MS UI stationary phase (J&W Scientific).

The injector temperature was 270°C, the purge flow rate was 20mL/min and the purge was turned on after 60 seconds. The gas flow rate through the column was 1mL/min, the column temperature was held at 70°C for 2 minutes, then increased by 40°C/min to 320°C, and held there for 2 minutes. The column effluent was introduced into the ion source of a Pegasus III time-of-flight mass spectrometer, GC/TOFMS (Leco Corp., St Joseph, MI, USA). The transfer line and the ion source temperatures were 250°C and 200°C, respectively. Ions were generated by a 70eV electron beam at an ionization current of 2.0mA, and 30spectra/s were recorded in the mass range m/z 50-800. The acceleration voltage was turned on after a solvent delay of 150 seconds. The detector voltage was 1500-2000V.

Targeted Data Processing

LC-MS

For LC-MS, all the data processing was performed using the Agilent Masshunter Profinder version B.08.00 (Agilent Technologies Inc., Santa Clara, CA, USA). For the targeted data processing, a pre-defined list of metabolites commonly found in plasma and serum were searched for using the Batch Targeted feature extraction in Masshunter Profinder. An-in-house LC-MS library built up by authentic standards run on the same system with the same chromatographic and mass-spec settings, were used for the targeted processing. The identification of the metabolites was based on MS, MSMS and retention time information. Multivariate statistical tests were performed to investigate the potential effect of run-order to the metabolite intensities (SIMCA v13.0.2, Umetrics, Umeå, Sweden).

GC-MS

For the GC-MS data, all non-processed MS-files from the metabolic analysis were exported from the ChromaTOF software in NetCDF format to MATLAB® R2016a (Mathworks, Natick, MA, USA), where all data pre-treatment procedures, such as base-line correction, chromatogram alignment, data compression and Multivariate Curve Resolution were performed using custom scripts. The extracted mass spectra were identified by comparisons of their retention index and mass spectra with libraries of retention time indices and mass spectra[7]. Mass spectra and retention index comparison was performed using the NIST MS 2.0 software. Annotation of mass spectra was based on reverse and forward searches in the library. Masses and ratio between masses indicative of a derivatized metabolite were especially notified. If the mass spectrum according to SMC's experience was with the highest probability indicative of a metabolite and the retention index between the sample and library for the suggested metabolite was ±5 (usually less than 3) the deconvoluted "peak" was annotated as an identification of a metabolite. Multivariate statistical tests were performed to investigate the potential effect of run-order to the metabolite intensities (SIMCA v13.0.2, Umetrics, Umeå, Sweden).

Solvents
Methanol, HPLC-grade was obtained from Fischer Scientific (Waltham, MA, USA). Chloroform, Suprasolv for GC was obtained from Merck (Darmstadt, Germany). Acetonitrile, HPLC-grade was obtained from Fischer Scientific (Waltham, MA, USA). 2-Propanol, HPLC-grade was obtained from VWR (Radnor, PA, USA). H2O, Milli-Q.

Reference and tuning standards
Purine, 4 µM, Agilent Technologies (Santa Clara, CA, USA). HP-0921 (Hexakis(1H,1H,3H-tetrafluoropropoxy)phosphazine), 1 µM, Agilent Technologies (Santa Clara, CA, USA). Calibrant, ESI-TOF, ESI-L Low Concentration Tuning Mix, Agilent Technologies (Santa Clara, CA, USA). HP-0321 (Hexamethoxyphosphazine), 0.1 mM, Agilent Technologies (Santa Clara, CA, USA).

Stable isotopes for internal standards

LC-MS internal standards
13C9-Phenylalanine, 13C3-Caffeine, D4-Cholic acid, D8-Arachidonic Acid, 13C9-Caffeic Acid were obtained from Sigma (St. Louis, MO, USA).

GC-MS internal standards
L-proline-13C5, alpha-ketoglutarate-13C4, myristic acid-13C3, cholesterol-D7 were obtained from Cil (Andover, MA, USA). Succinic acid-D4, salicylic acid-D6, L-glutamic acid-13C5,15N, putrescine-D4, hexadecanoic acid-13C4, D-glucose-13C6, D-sucrose-13C12 were obtained from Sigma (St. Louis, MO, USA).

Overview for metabolic profiling
Raw intensities of internal standards (IS) from serum and VAT differed from those of pancreatic islets, liver and muscle in LC-MS (Supplementary Figure S2). In GC-MS this applied only to VAT, while IS for serum were similar to the other tissues (Supplementary Figure S3). IS within VAT in LC-MS showed larger variability (18-30%) when compared to IS within other tissues (Supplementary Figure S2), albeit this did not apply to GC-MS (Supplementary Figure S3). The differences and variations in IS intensities were most likely due to the different metabolite extraction protocols applied to VAT, serum and the other tissues (Supplementary Note – Sample Preparation). Nevertheless, the relative standard deviation of the intensities in LC-MS was below 20% for all IS that ionize well in the respective mode. The difference between the theoretical and the experimental values for retention time and mass were within accepted limits for LC-MS (Supplementary Figures S6 and S7). Only arachidonic acid in LC-MS negative mode for serum deviated among IS (Supplementary Figures S6 and S7). This might be caused by another compound in serum eluting at the same time, potentially saturating the detector and as a consequence impairing the mass accuracy. Taken together this information suggested that despite a limited number of varying compound intensities, we would be able to proceed with the analysis after data transformation (cf. Methods - Data Transformation and Normalization).

An imperceptible number of samples appeared to deviate due to fluctuation of specific metabolites' intensities, different peak shapes or additional peaks on certain mass channels (e.g. Supplementary Figure S4 – Pancreatic Islets positive mode). However, this was a consistently negligible number across tissues (Supplementary Figures S4 and S5). The randomized running order of samples did not affect the structure of the samples (Supplementary Figures S4 and S6).

MoDentify
MoDentify is a computational tool that identifies modules of metabolites that are associated with the outcome, in this case T2D. In general, it takes advantage of multi-tissue metabolomics cohorts to increase the statistical power and to maximize the association of metabolites to the respective outcome. Specifically, it computes partial correlations of pairs of metabolites, while regressing out the effect of all the other metabolites present in the set. Strongly connected metabolites are then represented in a network that is used to identify the modules of metabolites through a score maximization function. Finally, the set of metabolites participating in these modules can be used to explore the associations of the outcome to metabolites or modules of metabolites.

We applied MoDentify to the unified set of metabolites for all tissues and their covariates[8]. We first asked MoDentify to compute Gaussian graphical models of metabolites and to generate a network of the correlated metabolites (FDR<5%) (Supplementary Figures S18 and S19)[9]. Next, we computed functional modules of metabolites choosing the notion of eigenmetabolites as the module representative (FDR<5%) that quantifies the correlation of the module to the outcome. Finally, the network was imported in Cytoscape and taxa of metabolites of interest (e.g. AAs, Lipids) were highlighted (Figure 4).

PATHWAY AND TAXONOMY ENRICHMENT ANALYSIS

We used the human metabolome database (HMDB)[10] identifiers of significant metabolites (Mann-Whitney U test permuted p-value≤0.1 or fold-change 50%) from each tissue to explore the classification, pathway and biological roles enrichment using MBROLE 2.0 (Supplementary Tables S4, S5 and S6)[11]. We used the full set of metabolites for homo sapiens as background.

MULTIVARIATE STATISTICAL ANALYSIS

We prepared datasets that contained samples in the rows and metabolites (variables) in the columns for each tissue. The last column was added to the dataset and contained the decision: ND for don-diabetes or T2D for type 2 diabetes. We averaged duplicate variables detected by different MS methods[12]. Next we ran Boruta with a different seed for each tissue (Supplementary Table S8)[13]. We restricted Boruta on 10000 iterations and forced it to record intermediate randomization results of the quality-control features in order to allow visual exploration of the selected features (Supplementary Figure S20).

RULE-BASED CLASSIFICATION

ROSETTA[14] is founded in the theory of rough sets[15]. It generates minimal *IF-THEN* rule-based models from decision tables where rows are objects (e.g. samples) and columns are pairs of measured variables (features, attributes) with their values (e.g. a metabolite and its level for the sample) while the last column is the outcome (also known as decision), here type 2 diabetes or Non-diabetes. A sample rule may be of the form:

IF metabolite-1 is DOWN AND metabolite-32 is UNCHANGED AND metabolite-230 is UP THEN outcome=T2D

The pairs are called descriptors.

Rule-based models belong to the family of so-called transparent machine learning models with decision trees[16] being another well-known member of this family. These models are legible and have the advantage of explicitly defining the outcome in the terms of variables and their values. Several statistical quality measures may be associated with rules and rule models. In this work we have used an R wrapper implementation[17] of the ROSETTA system.

IF-THEN rules are characterized by the left-hand support (LHS) and right-hand support (RHS). LHS stands for the number of objects (samples) that satisfy the IF part of the rule. RHS stands for the number of objects (samples) that satisfy the IF and the THEN part of the rule. However, given that there might be objects that satisfy the IF part of the rule (LHS) but their observed decision does not follow the one that the rule has (LHS≠RHS), we calculate rule accuracy (RA). RA stands for the ratio of RHS over LHS. The quality of each rule is additionally assessed by a p-value that is derived from a hypergeometric distribution.

Transparent models not only offer predictions and classification, but also they can be studied in detail since non-experts easily read rules. Another property of rule-based models that makes them attractive is the possibility of studying interactions between the descriptors, eg. pairs of metabolite and their levels associated to the type 2 diabetes outcome. This possibility is explored by the VisuNet rule network visualization package[18].

Since some problems in genomics may be computationally prohibitively expensive due to very high dimensionality (much fewer samples than variables (n<<p)), there is a need for methods that select the most informative set of variables that

best at predicting the outcome. This step, also known as feature or variable selection, was performed with Boruta (Supplementary Note - Multivariate Statistical Analysis)[13].

Here we used the set of features that Boruta marked as “Confirmed” or “Tentative” (Supplementary Figure S20). We next used these datasets and developed tissue-specific models. We aimed at exploring the combinatorics of sets of metabolites that predict Non-Diabetes or type 2 diabetes in various tissues. ROSETTA requires data to be discretized, and here applied equal frequency binning to split metabolite values into ranges containing an equal number of samples. ROSETTA was run in default settings except from the number of cross-validation to be performed that was set to 5 due to the limited number of samples, and the fallback classification class that was set to type 2 diabetes. The quality of the rule-based classifier was verified by classic machine learning metrics such as accuracy and receiver operating characteristic (ROC) area under the curve (AUC) (Supplementary Table S8).

VisuNet (http://bioinf.icm.uu.se/~visunet/index.php) is a package to display rule networks[18]. Nodes of the networks are descriptors and edges are put between the nodes that co-occur in a rule. Several numerical values may be associated to edges and nodes. The more often a pair of descriptors co-occurs in rules and the higher is the accuracy of these rules, the thicker is the edge. And if the descriptor occurs in many rules, the size of the node grows. Rule networks help discover which descriptors may be central in defining the outcome and which descriptors may interact.

In our study, VisuNet was provided with the set of statistically significant rules from ROSETTA (p-value < 0.1) and with the following settings: File format: Line-by-line; Minimum Accuracy: 0.1; Minimum Support: 1; Threshold (%): 100; Color of Nodes: Level of gene expression. Visualizations from VisuNet are shown in (Supplementary Figure S21).

SUPPLEMENTARY FIGURES

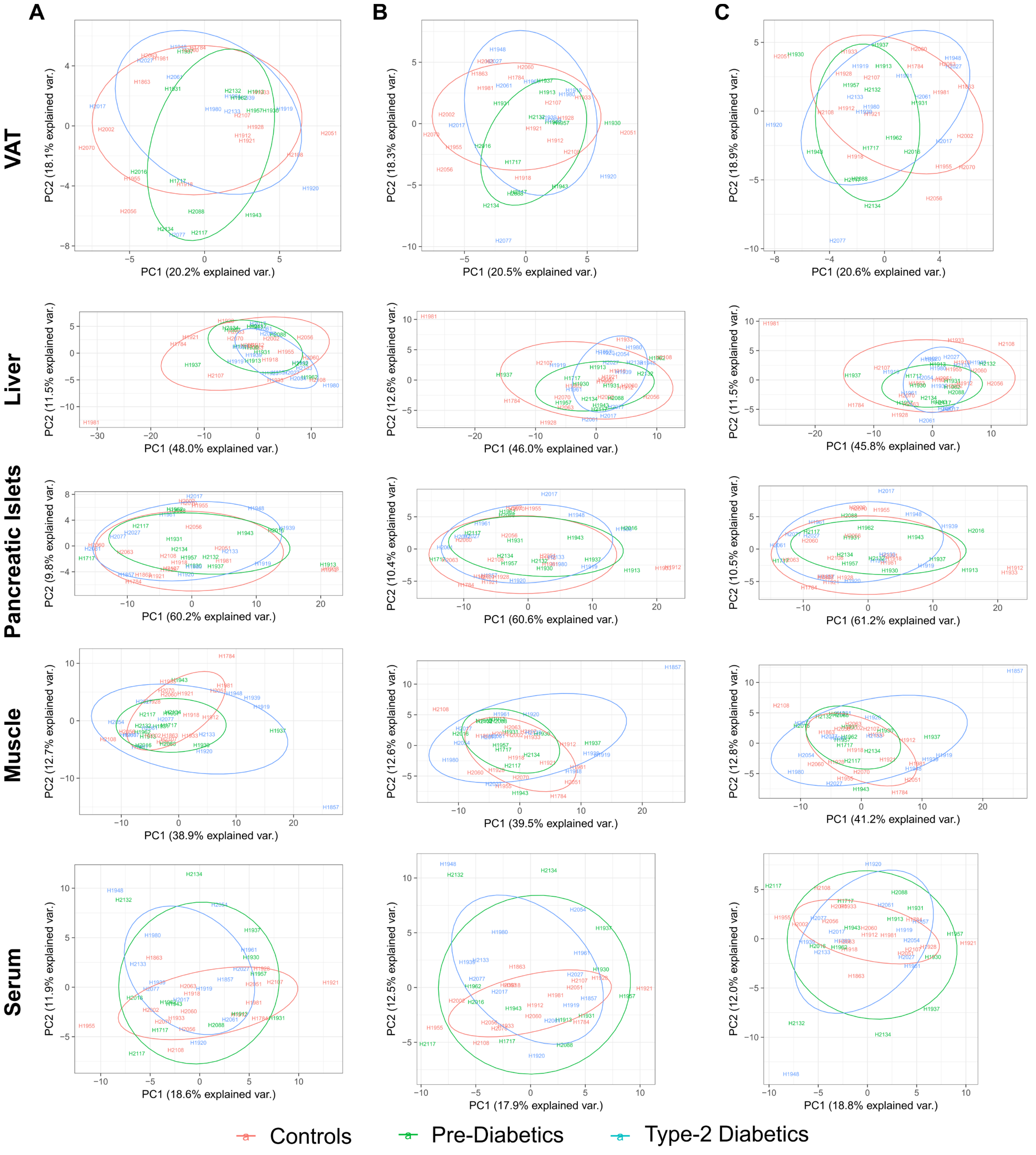

Supplementary Figure S1: Principle component analysis (PCA) plots for the 2 first principle components of $\log_2$ transformed and scaled to $\mu=0$ and $\sigma=1$ metabolite intensities for Controls, Pre-diabetes and Type-2 Diabetes. The five tissues are shown in the rows. **A)** Data not corrected for cofactors; **B)** Data corrected for BMI, Age, Gender and Sample Weight; **C)** Data corrected for BMI, Age, Gender, Sample Weight, GSIS and HbA1c.

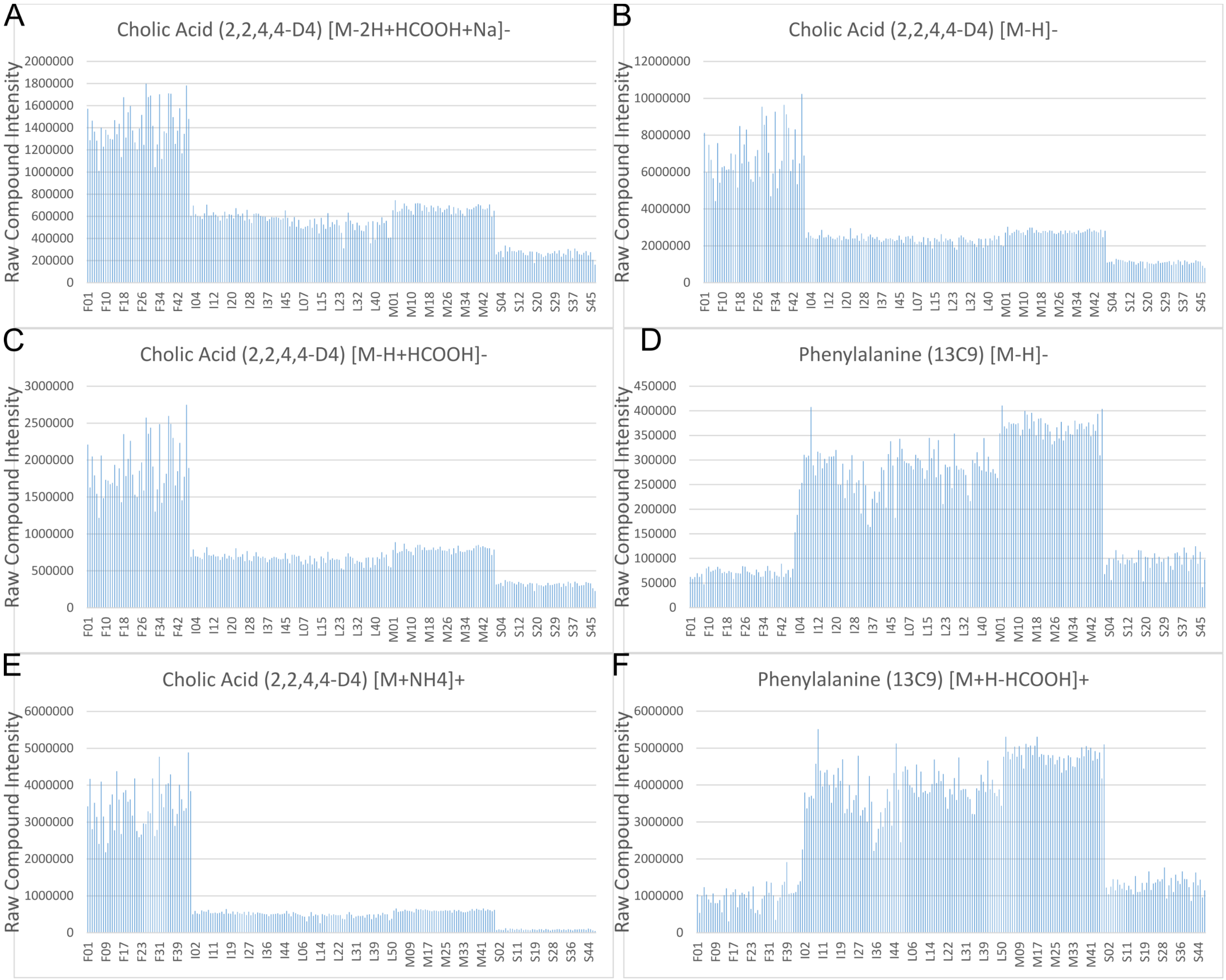

Supplementary Figure S2: Intensity (area) for various internal standards (name of compound mentioned in each plot) in all tissues for LC-MS in **A-D)** negative and **E-F)** positive mode. Note that VAT is abbreviated with "F", pancreatic islets with "I", liver with "L", muscle with "M" and serum with "S".

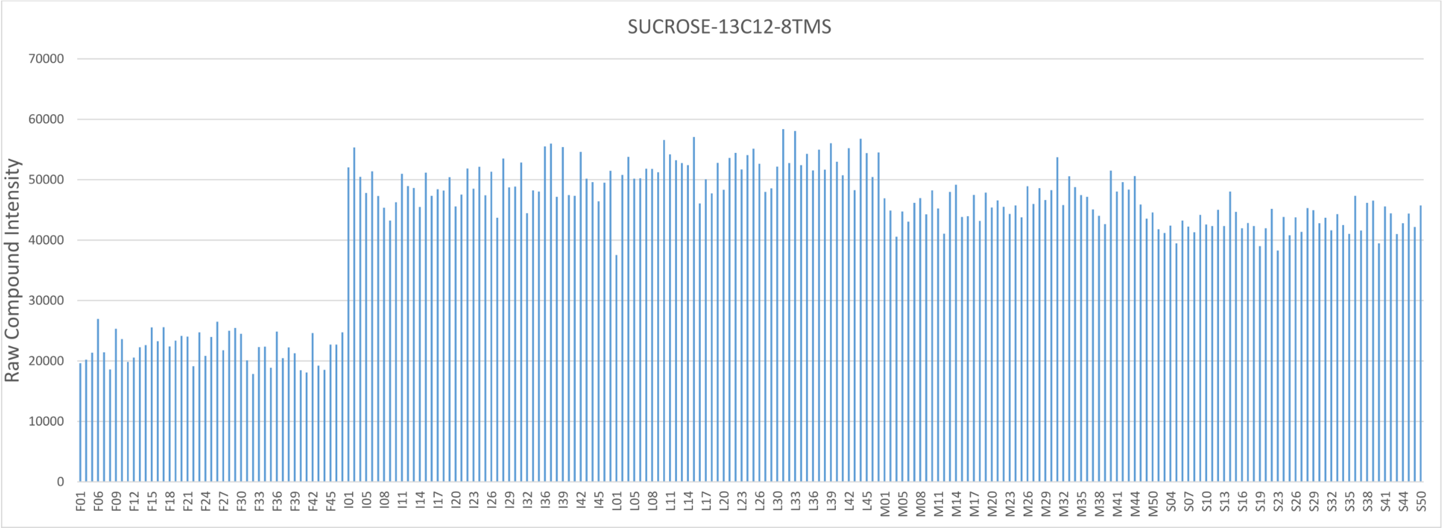

Supplementary Figure S3: Intensity (area) for internal standards (Sucrose-13C12-8TMS) for GC-MS. Note that VAT is abbreviated with "F", pancreatic islets with "I", liver with "L", muscle with "M" and serum with "P".

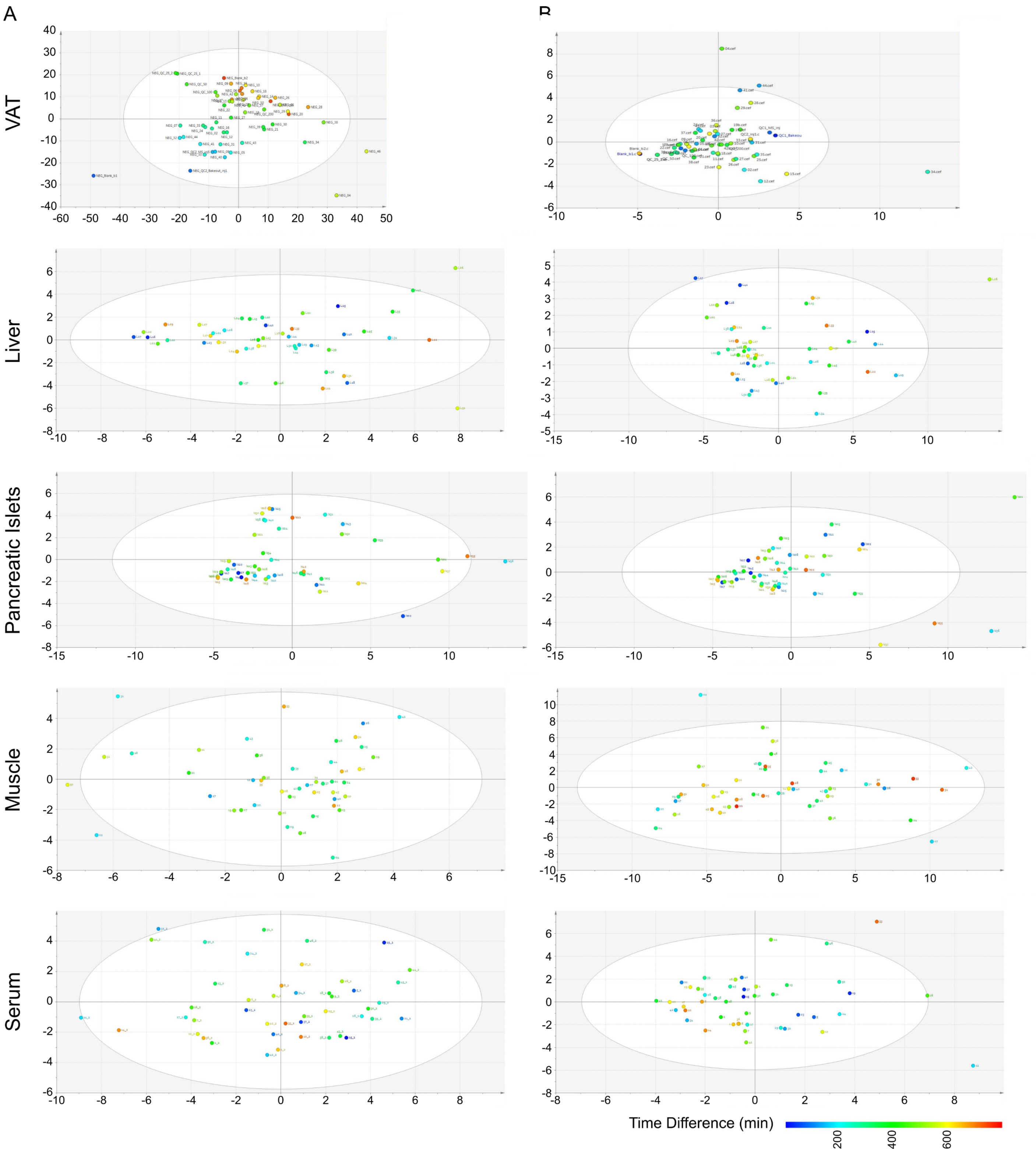

Supplementary Figure S4: Principle component analysis (PCA) plots for the two first principle components of raw metabolite intensities in LC-MS for each tissue (rows) color-coded for run-order (Supplementary Methods – Metabolic Profiling). **A)** LC-MS negative mode; **B)** LC-MS positive mode.

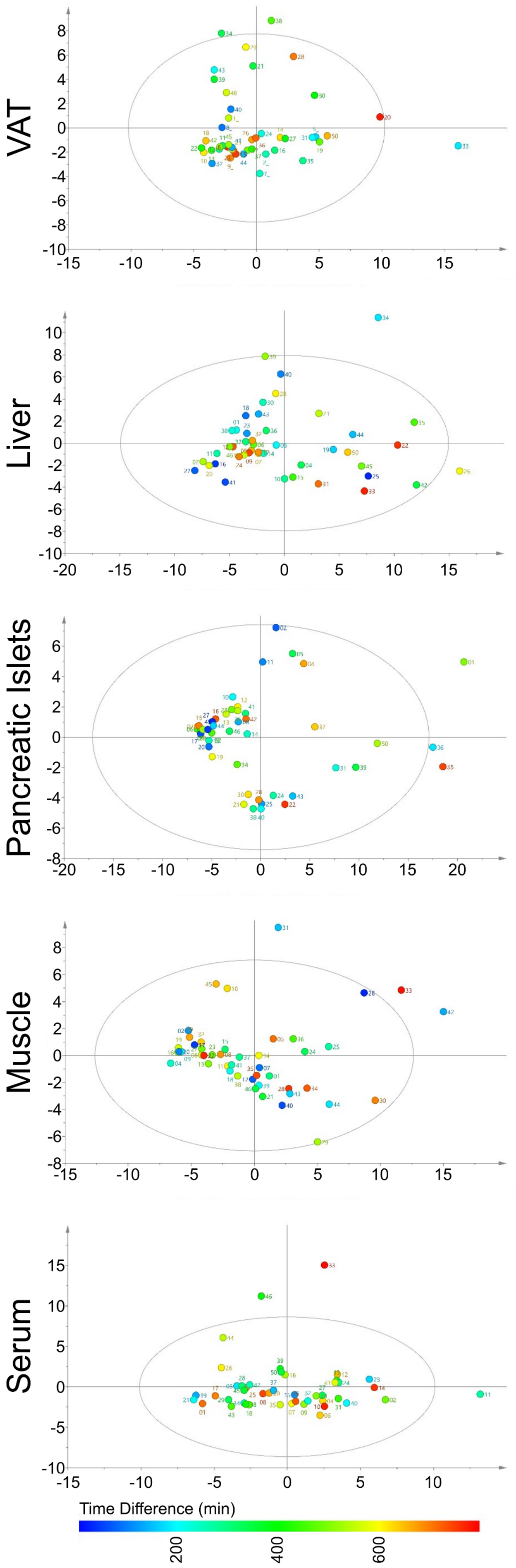

VAT
Liver
Pancreatic Islets
Muscle
Serum
Time Difference (min)
200
400
600

Supplementary Figure S5: Principle component analysis (PCA) plots for the two first principle components of raw metabolite intensities in GC-MS for each tissue (rows) color-coded for run-order (Supplementary Methods – Metabolic Profiling).

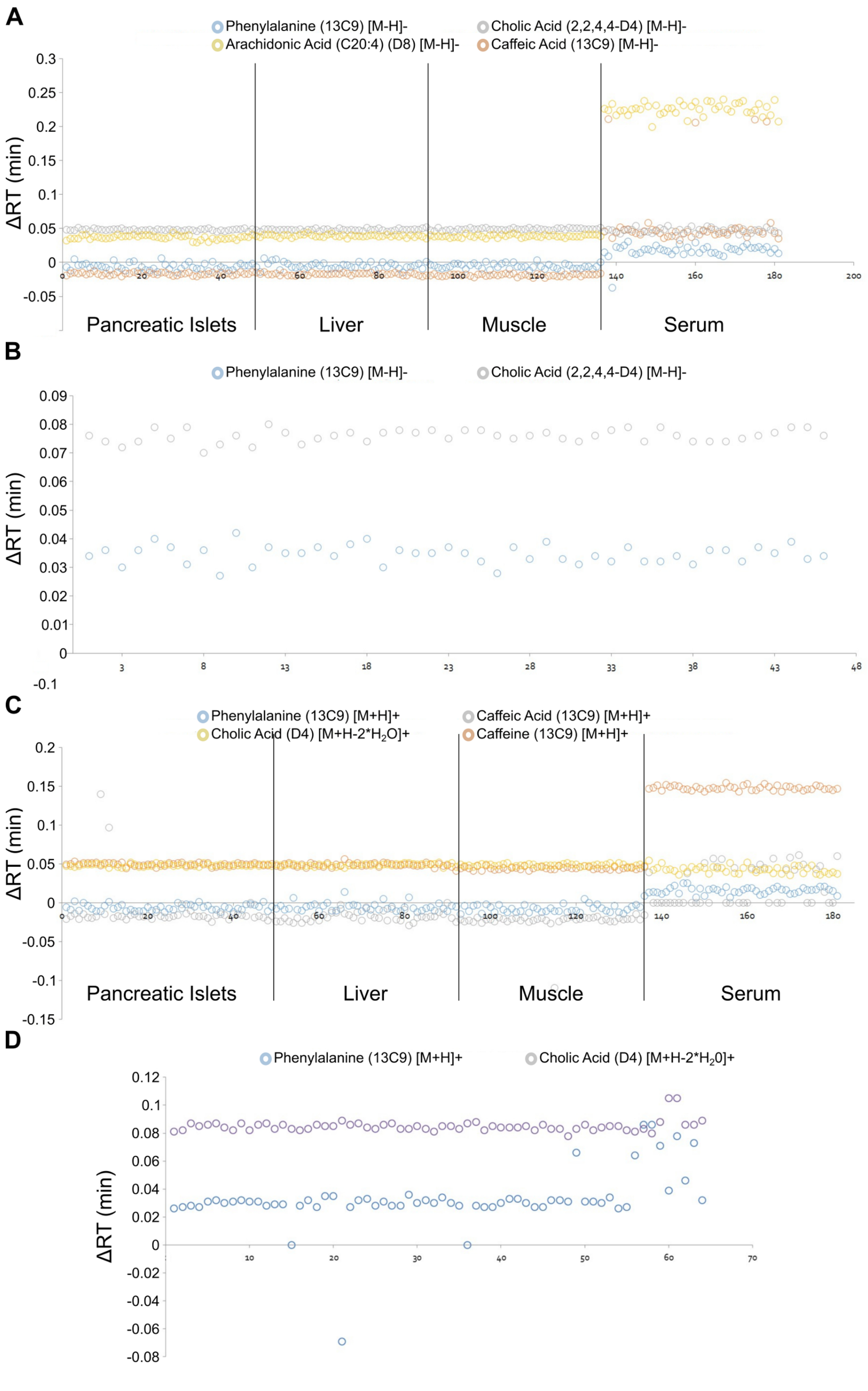

Supplementary Figure S6: Difference (delta – Δ) between theoretical and observed retention time in minutes for internal standards in LC-MS. **A)** and **B)** negative mode; **C)** and **D)** positive mode. **B)** and **D)** are for VAT, while **A)** and **C)** for the other four tissues (liver, pancreatic islets, muscle and serum).

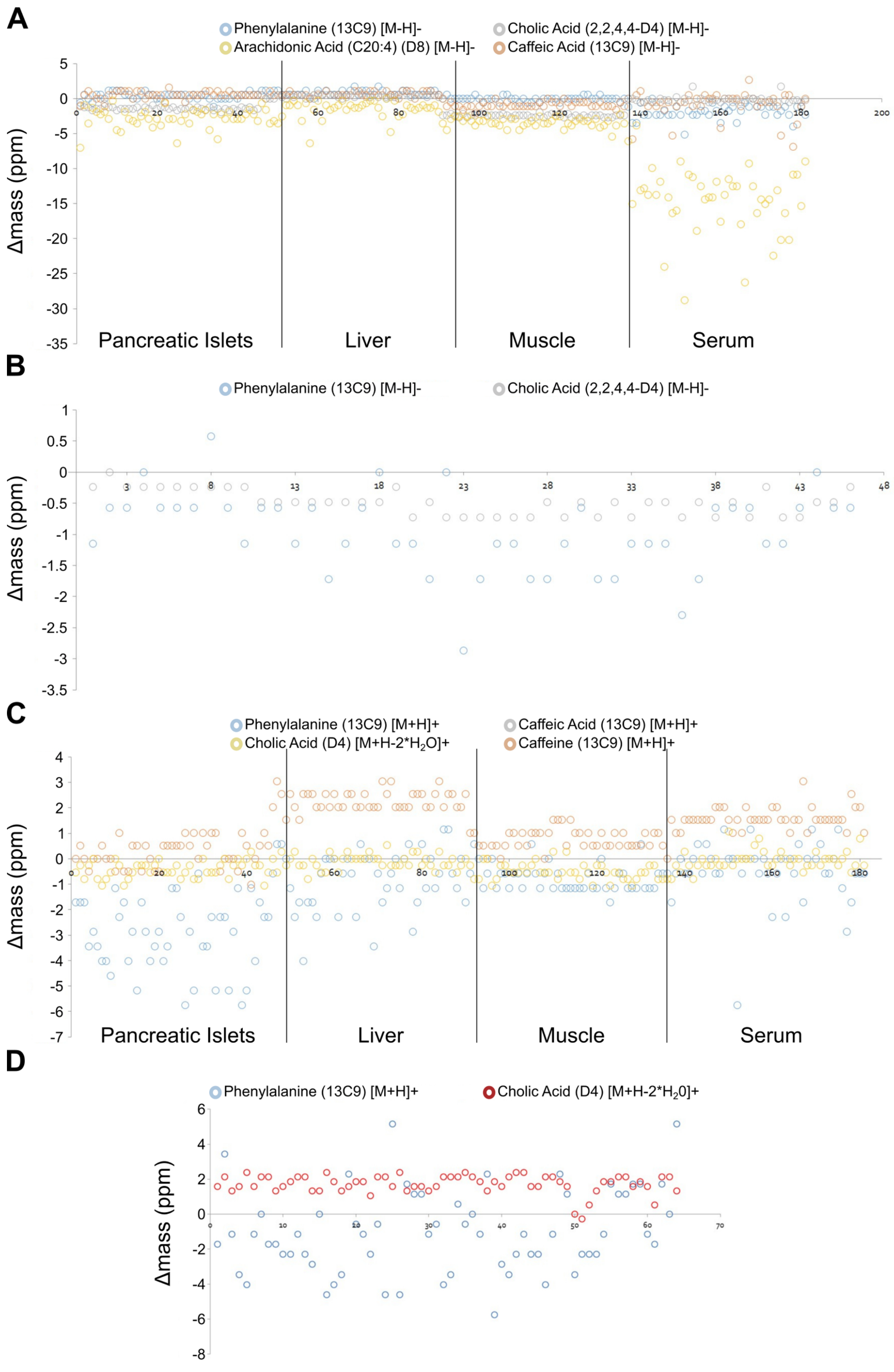

Supplementary Figure S7: Difference (delta – Δ) between theoretical and observed mass in parts per million for internal standards in LC-MS. **A)** and **B)** negative mode; **C)** and **D)** positive mode. **B)** and **D)** are for VAT, while **A)** and **C)** for the other four tissues (liver, pancreatic islets, muscle and serum).

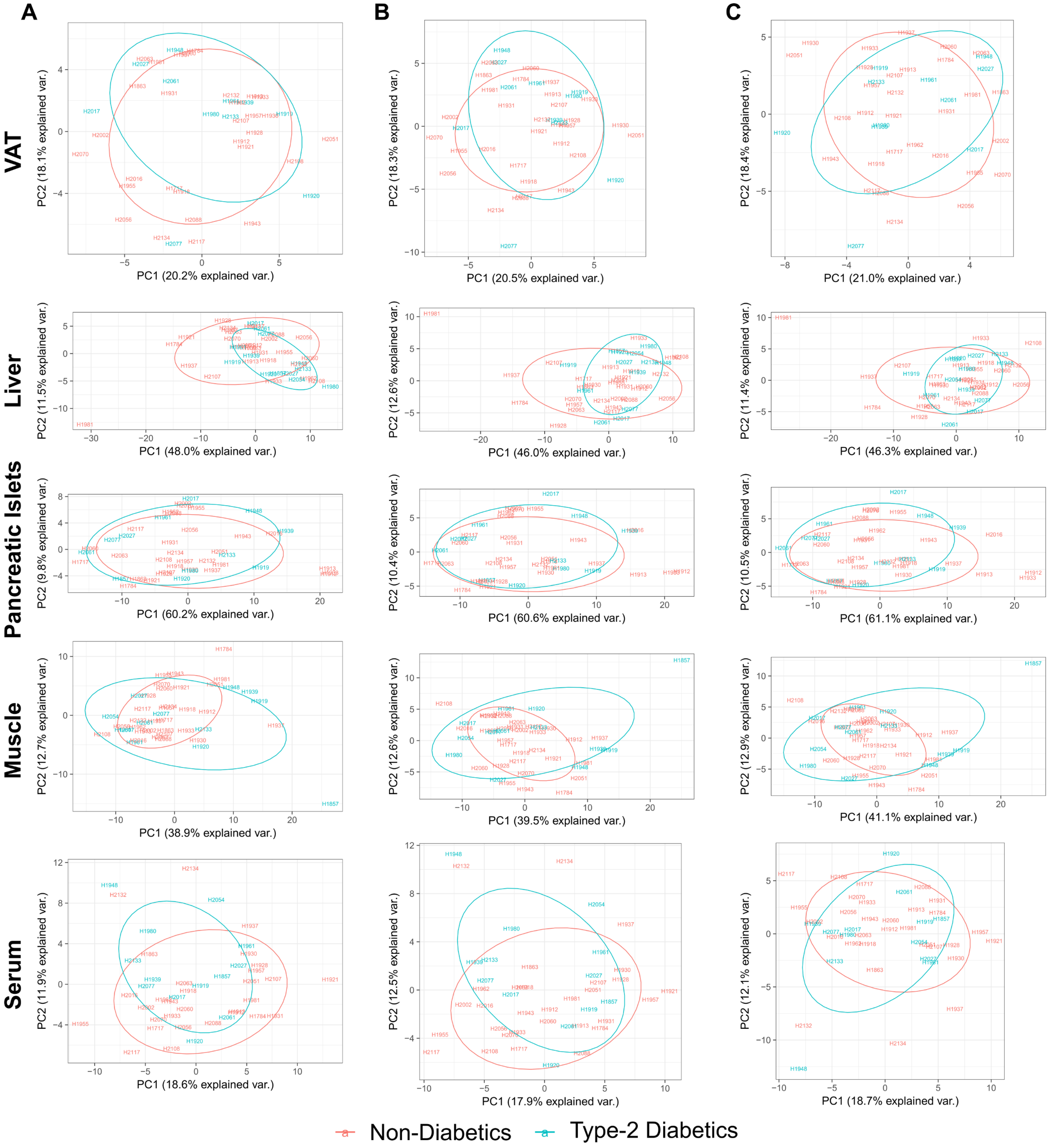

Supplementary Figure S8: Principle component analysis (PCA) plots for the 2 first principle components of log_2 transformed and scaled to $\mu=0$ and $\sigma=1$ metabolite intensities for Non-diabetes and Type-2 Diabetes. The five tissues are shown in the rows. **A)** Data not corrected for cofactors; **B)** Data corrected for BMI, Age, Gender and Sample Weight; **C)** Data corrected for BMI, Age, Gender, Sample Weight, GSIS and HbA1c.

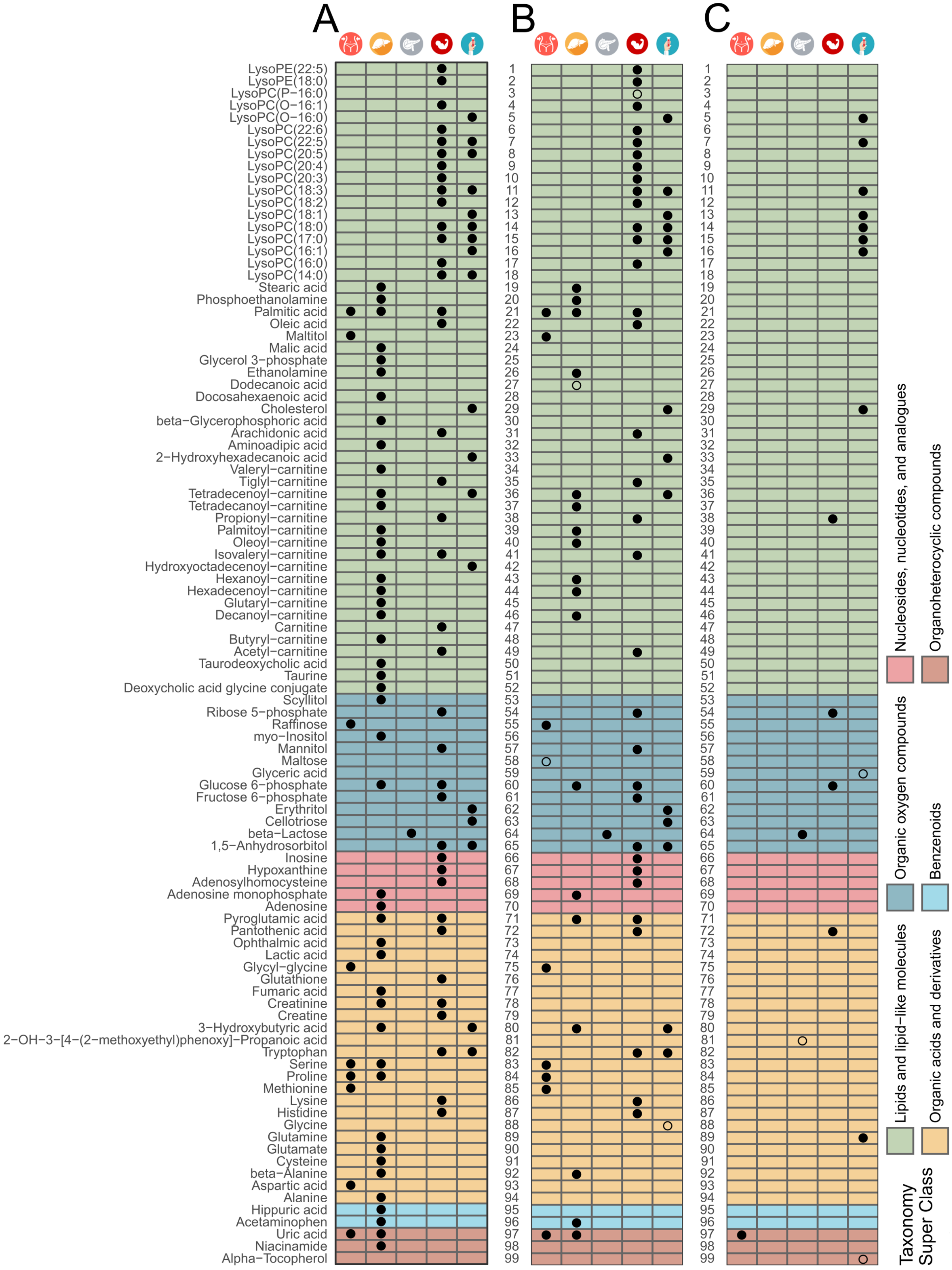
A
B
C
LysoPE(22:5)
LysoPE(18:0)
LysoPC(P−16:0)
LysoPC(O−16:1)
LysoPC(O−16:0)
LysoPC(22:6)
LysoPC(22:5)
LysoPC(20:5)
LysoPC(20:4)
LysoPC(20:3)
LysoPC(18:3)
LysoPC(18:2)
LysoPC(18:1)
LysoPC(18:0)
LysoPC(17:0)
LysoPC(16:1)
LysoPC(16:0)
LysoPC(14:0)
Stearic acid
Phosphoethanolamine
Palmitic acid
Oleic acid
Maltitol
Malic acid
Glycerol 3−phosphate
Ethanolamine
Dodecanoic acid
Docosahexaenoic acid
Cholesterol
beta−Glycerophosphoric acid
Arachidonic acid
Aminoadipic acid
2−Hydroxyhexadecanoic acid
Valeryl−carnitine
Tiglyl−carnitine
Tetradecenoyl−carnitine
Tetradecanoyl−carnitine
Propionyl−carnitine
Palmitoyl−carnitine
Oleoyl−carnitine
Isovaleryl−carnitine
Hydroxyoctadecenoyl−carnitine
Hexanoyl−carnitine
Hexadecenoyl−carnitine
Glutaryl−carnitine
Decanoyl−carnitine
Carnitine
Butyryl−carnitine
Acetyl−carnitine
Taurodeoxycholic acid
Taurine
Deoxycholic acid glycine conjugate
Scyllitol
Ribose 5−phosphate
Raffinose
myo−Inositol
Mannitol
Maltose
Glyceric acid
Glucose 6−phosphate
Fructose 6−phosphate
Erythritol
Cellotriose
beta−Lactose
1,5−Anhydrosorbitol
Inosine
Hypoxanthine
Adenosylhomocysteine
Adenosine monophosphate
Adenosine
Pyroglutamic acid
Pantothenic acid
Ophthalmic acid
Lactic acid
Glycyl−glycine
Glutathione
Fumaric acid
Creatinine
Creatine
3−Hydroxybutyric acid
2−OH−3−[4−(2−methoxyethyl)phenoxy]−Propanoic acid
Tryptophan
Serine
Proline
Methionine
Lysine
Histidine
Glycine
Glutamine
Glutamate
Cysteine
beta−Alanine
Aspartic acid
Alanine
Hippuric acid
Acetaminophen
Uric acid
Niacinamide
Alpha−Tocopherol
Taxonomy Super Class
Lipids and lipid−like molecules
Organic acids and derivatives
Organic oxygen compounds
Benzenoids
Nucleosides, nucleotides, and analogues
Organoheterocyclic compounds

Supplementary Figure S9: Statistically significant metabolites when comparing non-diabetes to type 2 diabetes. Significance was computed for $\log_2$ scaled to $\mu=0$ and $\sigma=1$ metabolite intensities. Rows represent metabolites and columns tissues. Color coding is based on manually curated classification of the metabolites based on HMDB's Super Class annotation[10]. A filled black dot states statistical significance (Mann-Whitney U test permuted p-value<0.1; Methods - Statistical analysis), while the empty dot shows new metabolites that appeared after correcting for covariates. **A)** Metabolite intensities without covariate correction; **B)** Metabolite intensities corrected for BMI, Age, Gender and Sample Weight; **C)** Metabolite intensities corrected for BMI, Age, Gender, Sample Weight, HbA1c and GSIS.

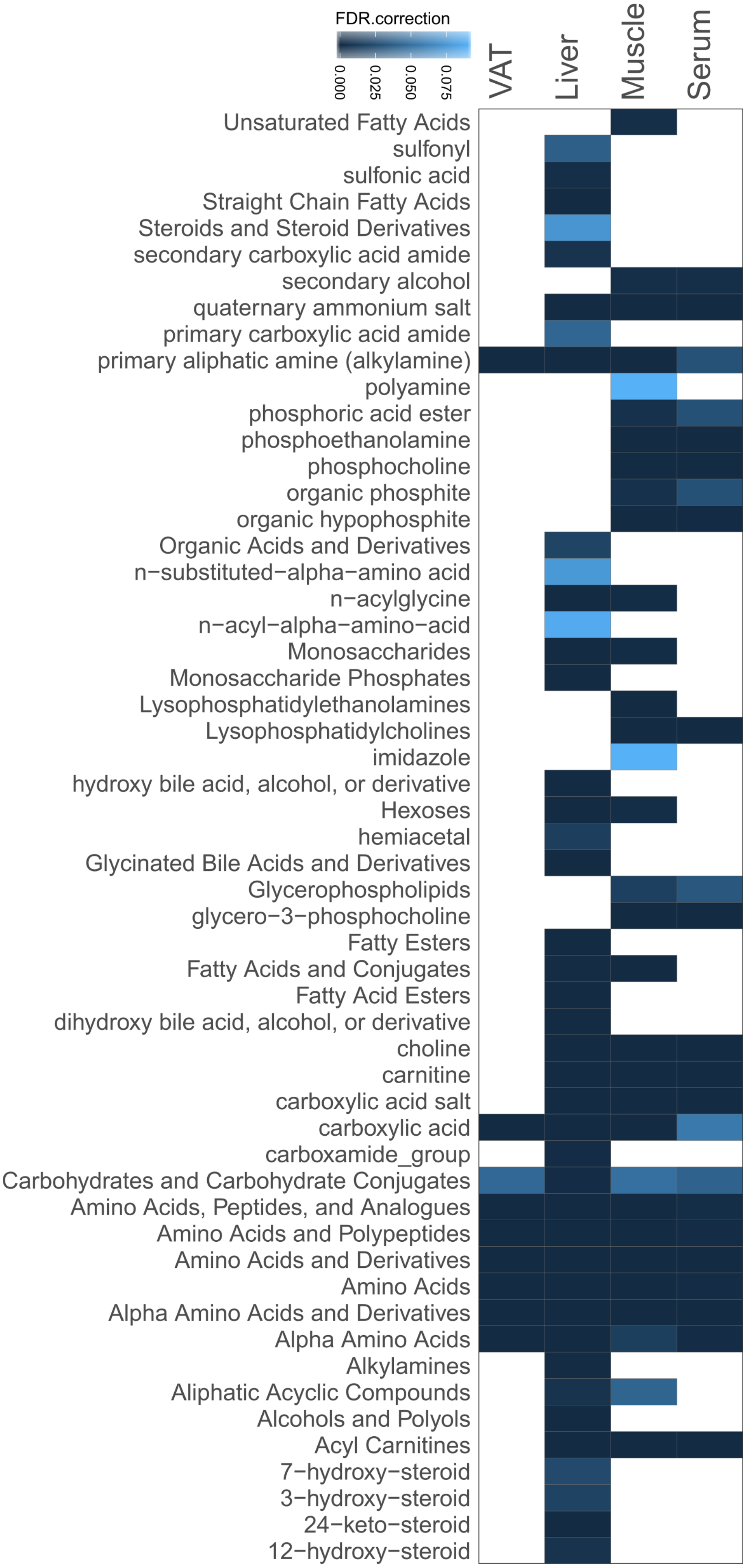
FDR.correction
0.000
0.025
0.050
0.075
VAT
Liver
Muscle
Serum
Unsaturated Fatty Acids
sulfonyl
sulfonic acid
Straight Chain Fatty Acids
Steroids and Steroid Derivatives
secondary carboxylic acid amide
secondary alcohol
quaternary ammonium salt
primary carboxylic acid amide
primary aliphatic amine (alkylamine)
polyamine
phosphoric acid ester
phosphoethanolamine
phosphocholine
organic phosphite
organic hypophosphite
Organic Acids and Derivatives
n−substituted−alpha−amino acid
n−acylglycine
n−acyl−alpha−amino−acid
Monosaccharides
Monosaccharide Phosphates
Lysophosphatidylethanolamines
Lysophosphatidylcholines
imidazole
hydroxy bile acid, alcohol, or derivative
Hexoses
hemiacetal
Glycinated Bile Acids and Derivatives
Glycerophospholipids
glycero−3−phosphocholine
Fatty Esters
Fatty Acids and Conjugates
Fatty Acid Esters
dihydroxy bile acid, alcohol, or derivative
choline
carnitine
carboxylic acid salt
carboxylic acid
carboxamide_group
Carbohydrates and Carbohydrate Conjugates
Amino Acids, Peptides, and Analogues
Amino Acids and Polypeptides
Amino Acids and Derivatives
Amino Acids
Alpha Amino Acids and Derivatives
Alpha Amino Acids
Alkylamines
Aliphatic Acyclic Compounds
Alcohols and Polyols
Acyl Carnitines
7−hydroxy−steroid
3−hydroxy−steroid
24−keto−steroid
12−hydroxy−steroid

Supplementary Figure S10: Metabolic taxa (classes) enriched in various tissues. Taxa enrichment was obtained from MBROLE2.0[11] for significant metabolites in the corresponding tissue (Methods - Statistical analysis; Supplementary Note - Pathway and taxonomy enrichment analysis). Color-coding is based on FDR corrected p-values (Supplementary Table S3).

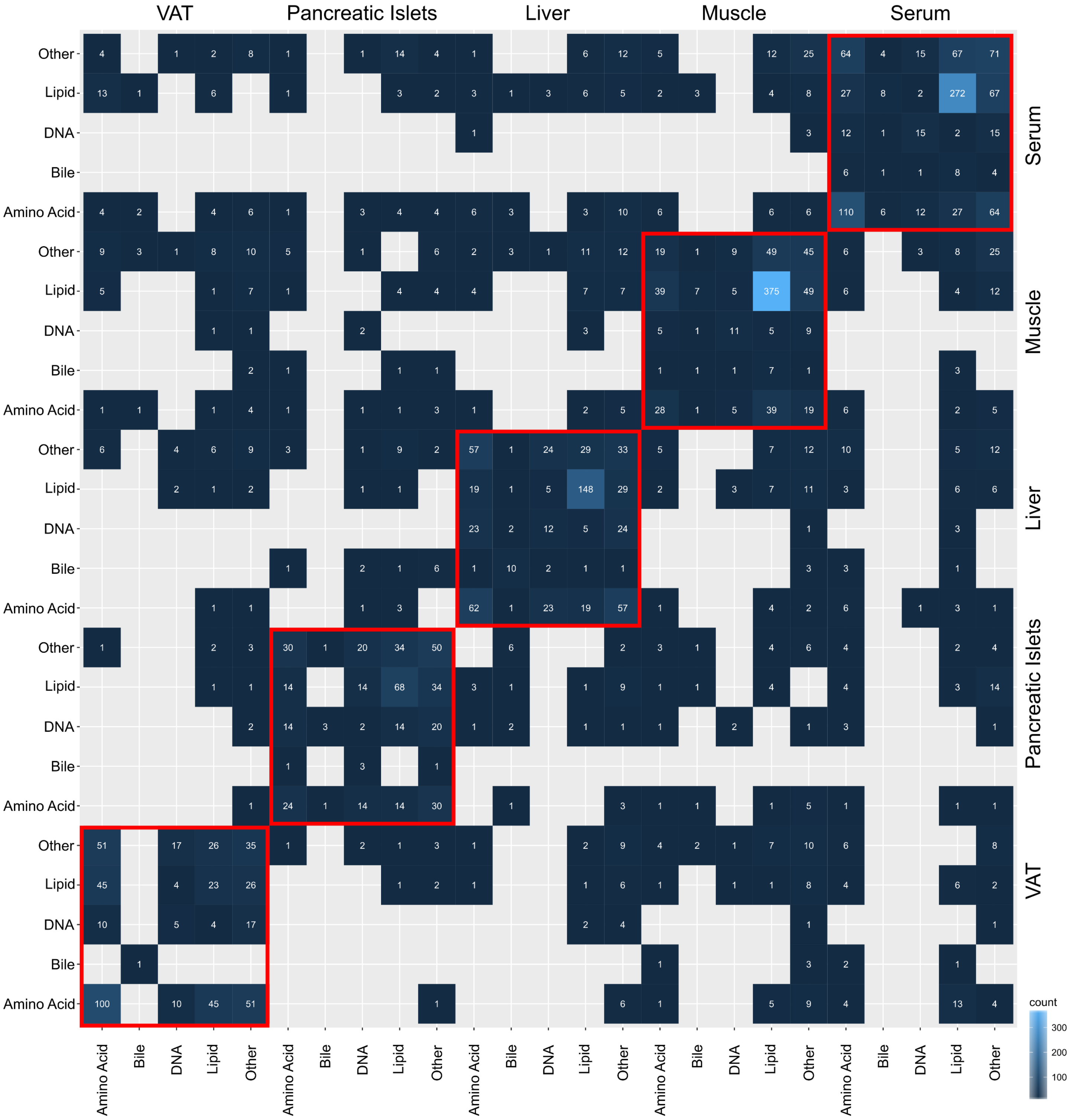

Supplementary Figure S11: MoDentify correlation matrix for manually annotated classes of metabolites for five metabolic tissues (rows and columns) (Supplementary Note - MoDentify)[8]. Classes belonging to the same tissue are marked in red boxes. The count of metabolite pairs correlated within the manually-annotated class (rows and columns) are noted in the matrix cells. The color intensity in the heatmap is representative of the number of the correlated compounds within the class.

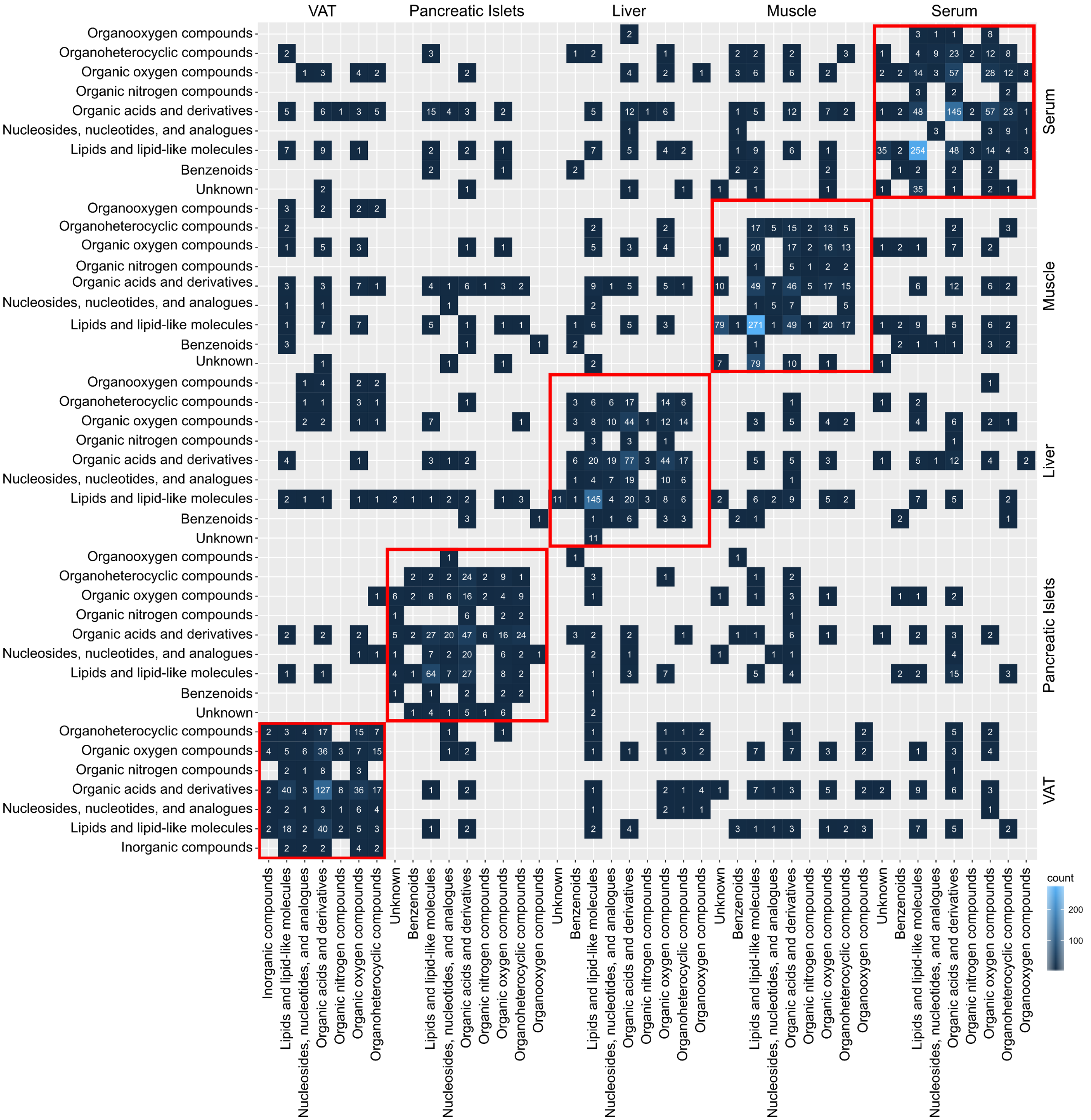

Supplementary Figure S12: MoDentify correlation matrix for metabolites' Super Classes annotated from HMDB for five metabolic tissues (rows and columns) (Supplementary Note - MoDentify)[8]. Super classes belonging to the same tissues are marked in red boxes. The count of metabolite pairs correlated within the HMDB-annotated class (rows and columns) are noted in the matrix cells. The color intensity in the heatmap is representative of the number of the correlated compounds within the class.

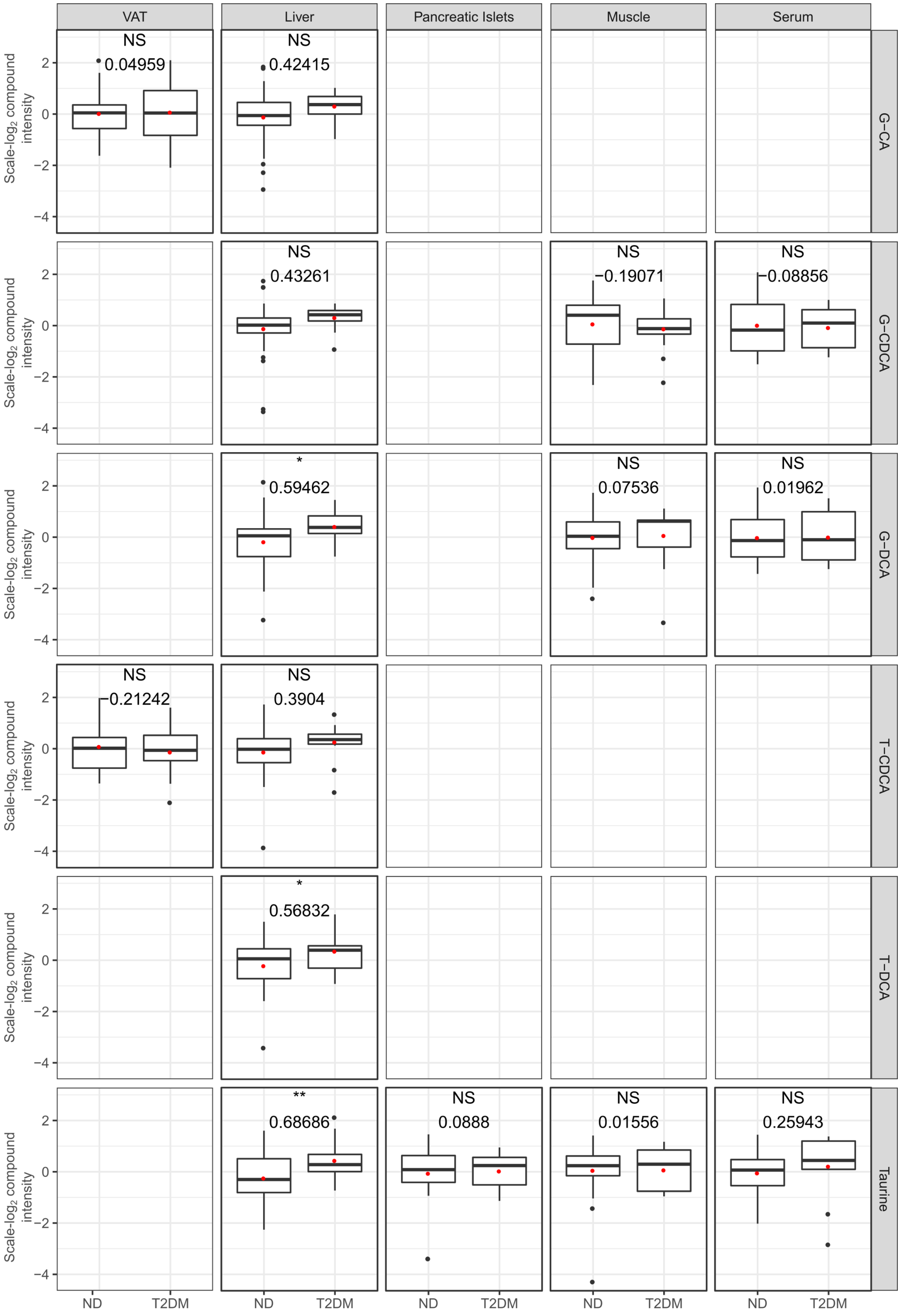

VAT
Liver
Pancreatic Islets
Muscle
Serum
G–CA
G–CDCA
G–DCA
T–CDCA
T–DCA
Taurine
Scale-log2 compound intensity
NS
0.04959
NS
0.42415
NS
0.43261
NS
−0.19071
NS
−0.08856
*
0.59462
NS
0.07536
NS
0.01962
NS
−0.21242
NS
0.3904
*
0.56832
**
0.68686
NS
0.0888
NS
0.01556
NS
0.25943
ND
T2DM

Supplementary Figure S13: Levels of bile acids across five metabolic tissues. Columns show tissues and rows metabolites. The text in each plot shows statistical significance (Mann-Whitney U test permuted p-value) (p-value>0.1 NS; 0.1≥p-value>0.05 *; 0.05≥p-value>0.01 **; 0.01≥p-value>0.001 ***; 0.001≥p-value ****) between Non-diabetes (ND) and type 2 diabetes (Methods – Statistical analysis). The second line of the text in each plot shows the mean fold-change. The red dot signifies the mean value of the group. Abbreviations: CA = Cholic acid; DCA = Deoxycholic acid; CDCA = Chenodeoxycholic acid; G/T = glycine/taurine conjugates. Empty plots mean that the metabolite was not detected in the corresponding tissue.

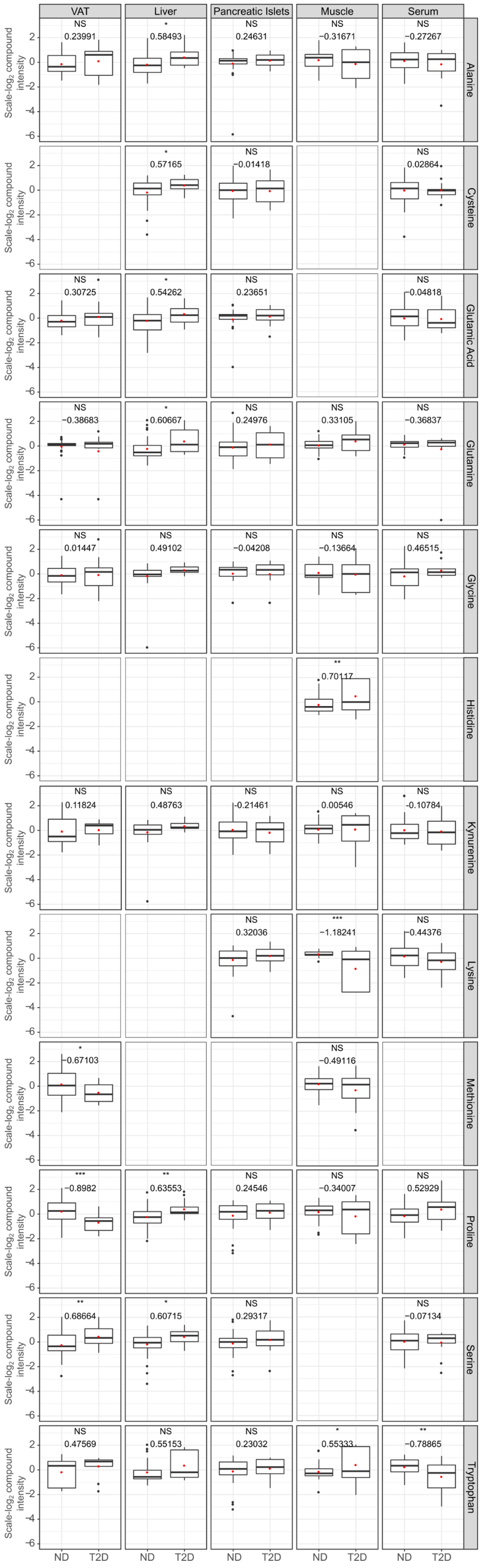

VAT
Liver
Pancreatic Islets
Muscle
Serum
Scale-log2 compound intensity
Alanine
NS 0.23991
* 0.58493
NS 0.24631
NS −0.31671
NS −0.27267
Cysteine
* 0.57165
NS −0.01418
NS 0.02864
Glutamic Acid
NS 0.30725
* 0.54262
NS 0.23651
NS −0.04818
Glutamine
NS −0.38683
* 0.60667
NS 0.24976
NS 0.33105
NS −0.36837
Glycine
NS 0.01447
NS 0.49102
NS −0.04208
NS −0.13664
NS 0.46515
Histidine
** 0.70117
Kynurenine
NS 0.11824
NS 0.48763
NS −0.21461
NS 0.00546
NS −0.10784
Lysine
NS 0.32036
*** −1.18241
NS −0.44376
Methionine
* −0.67103
NS −0.49116
Proline
*** −0.8982
** 0.63553
NS 0.24546
NS −0.34007
NS 0.52929
Serine
** 0.68664
* 0.60715
NS 0.29317
NS −0.07134
Tryptophan
NS 0.47569
NS 0.55153
NS 0.23032
* 0.55333
** −0.78865
ND
T2D

Supplementary Figure S14: Levels of selected amino acids (AAs) and products of them (Alanine, Glutamine, Glutamate/Glutamic Acid, Glycine, Proline, Serine, Histidine, Kynurenine, Lysine, Methionine, Cysteine and Tryptophan). Columns show tissues and rows metabolites. The text shows statistical significance (Mann-Whitney U test permuted p-value) (p-value>0.1 NS; 0.1≥p-value>0.05 *; 0.05≥p-value>0.01 **; 0.01≥p-value>0.001 ***; 0.001≥p-value ****) between Non-diabetes (ND) and type 2 diabetes (Methods – Statistical analysis). The second line of the text shows the mean fold-change. The red dot signifies the mean value of the group.

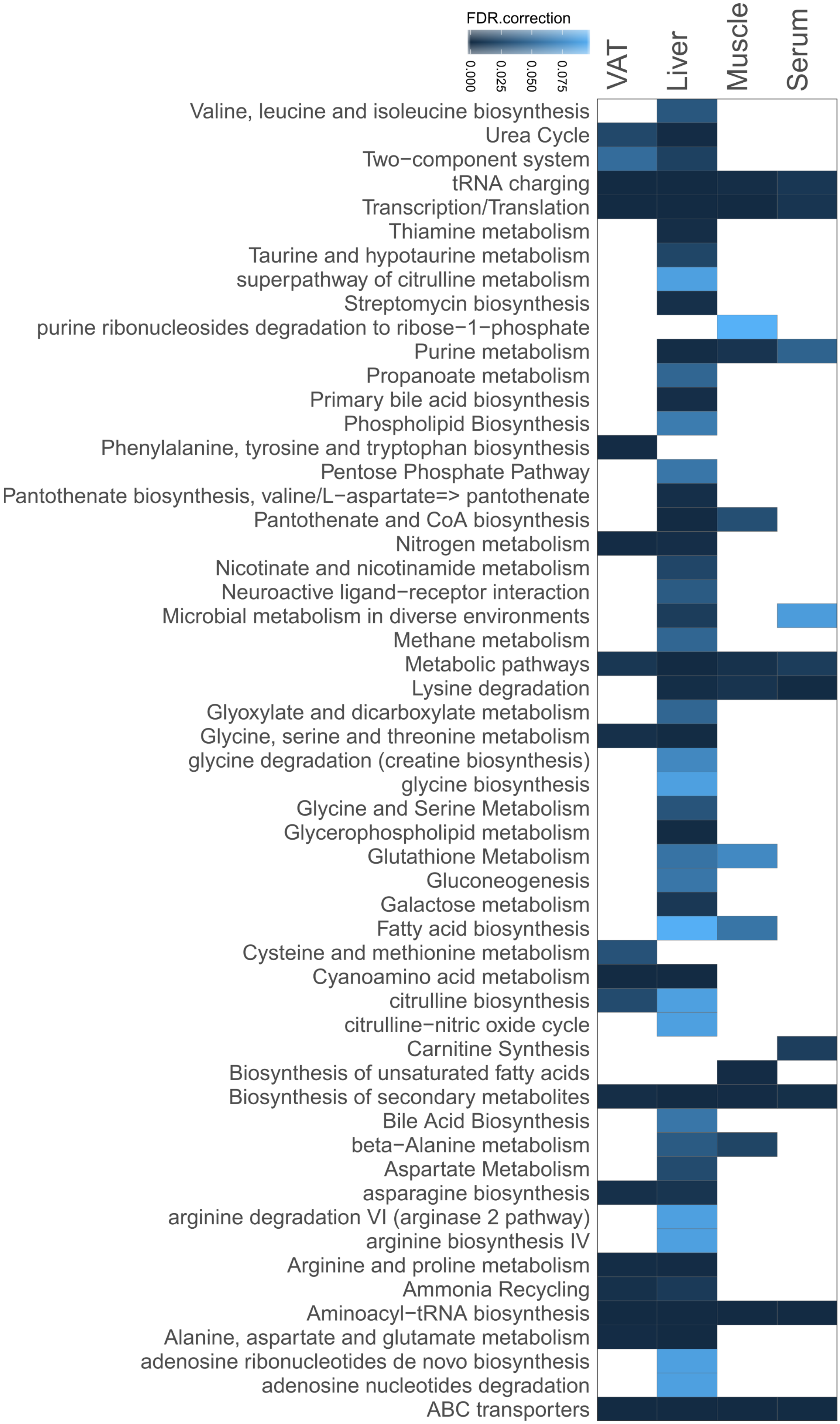
FDR.correction
0.000
0.025
0.050
0.075
VAT
Liver
Muscle
Serum
Valine, leucine and isoleucine biosynthesis
Urea Cycle
Two−component system
tRNA charging
Transcription/Translation
Thiamine metabolism
Taurine and hypotaurine metabolism
superpathway of citrulline metabolism
Streptomycin biosynthesis
purine ribonucleosides degradation to ribose−1−phosphate
Purine metabolism
Propanoate metabolism
Primary bile acid biosynthesis
Phospholipid Biosynthesis
Phenylalanine, tyrosine and tryptophan biosynthesis
Pentose Phosphate Pathway
Pantothenate biosynthesis, valine/L−aspartate=> pantothenate
Pantothenate and CoA biosynthesis
Nitrogen metabolism
Nicotinate and nicotinamide metabolism
Neuroactive ligand−receptor interaction
Microbial metabolism in diverse environments
Methane metabolism
Metabolic pathways
Lysine degradation
Glyoxylate and dicarboxylate metabolism
Glycine, serine and threonine metabolism
glycine degradation (creatine biosynthesis)
glycine biosynthesis
Glycine and Serine Metabolism
Glycerophospholipid metabolism
Glutathione Metabolism
Gluconeogenesis
Galactose metabolism
Fatty acid biosynthesis
Cysteine and methionine metabolism
Cyanoamino acid metabolism
citrulline biosynthesis
citrulline−nitric oxide cycle
Carnitine Synthesis
Biosynthesis of unsaturated fatty acids
Biosynthesis of secondary metabolites
Bile Acid Biosynthesis
beta−Alanine metabolism
Aspartate Metabolism
asparagine biosynthesis
arginine degradation VI (arginase 2 pathway)
arginine biosynthesis IV
Arginine and proline metabolism
Ammonia Recycling
Aminoacyl−tRNA biosynthesis
Alanine, aspartate and glutamate metabolism
adenosine ribonucleotides de novo biosynthesis
adenosine nucleotides degradation
ABC transporters

Supplementary Figure S15: Metabolic pathways enriched in various tissues. Pathway enrichment was obtained from MBROLE2.0[11] for significant metabolites in the corresponding tissue (Methods - Statistical analysis; Supplementary Note - Pathway and taxonomy enrichment analysis). Color-coding is based on FDR corrected p-values (Supplementary Table S4).

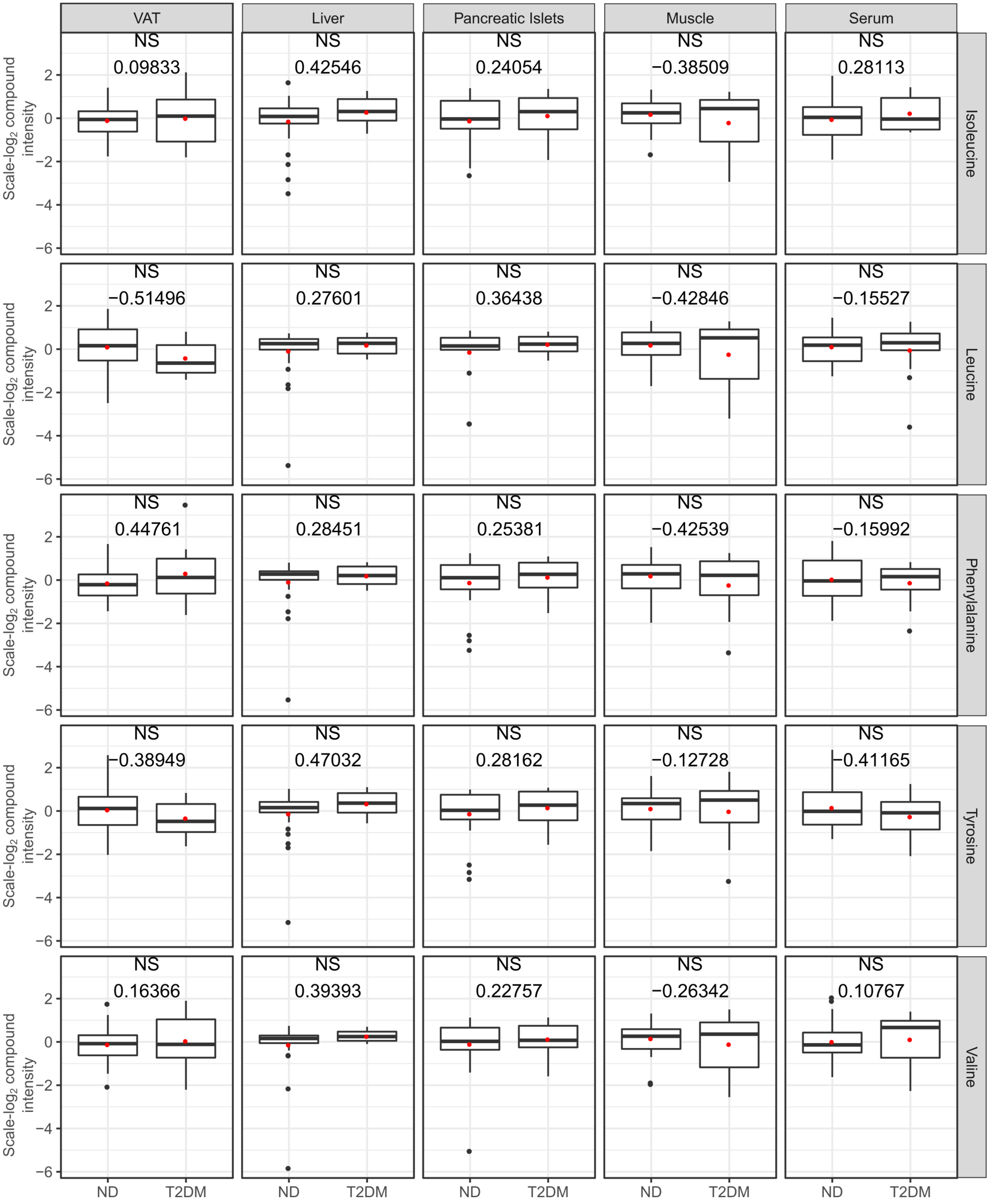

Supplementary Figure S16: Levels of branch-chain amino acids (BCAAs) and aromatic amino acids (AAAs). Columns show tissues and rows metabolites. The text shows statistical significance (Mann-Whitney U test permuted p-value) (p-

value>0.1 NS; 0.1≥p-value>0.05 *; 0.05≥p-value>0.01 **; 0.01≥p-value>0.001 ***; 0.001≥p-value ****) between Non-diabetes (ND) and type 2 diabetes (Methods – Statistical analysis). The second line of the text shows the mean fold change. The red dot signifies the mean value of the group.

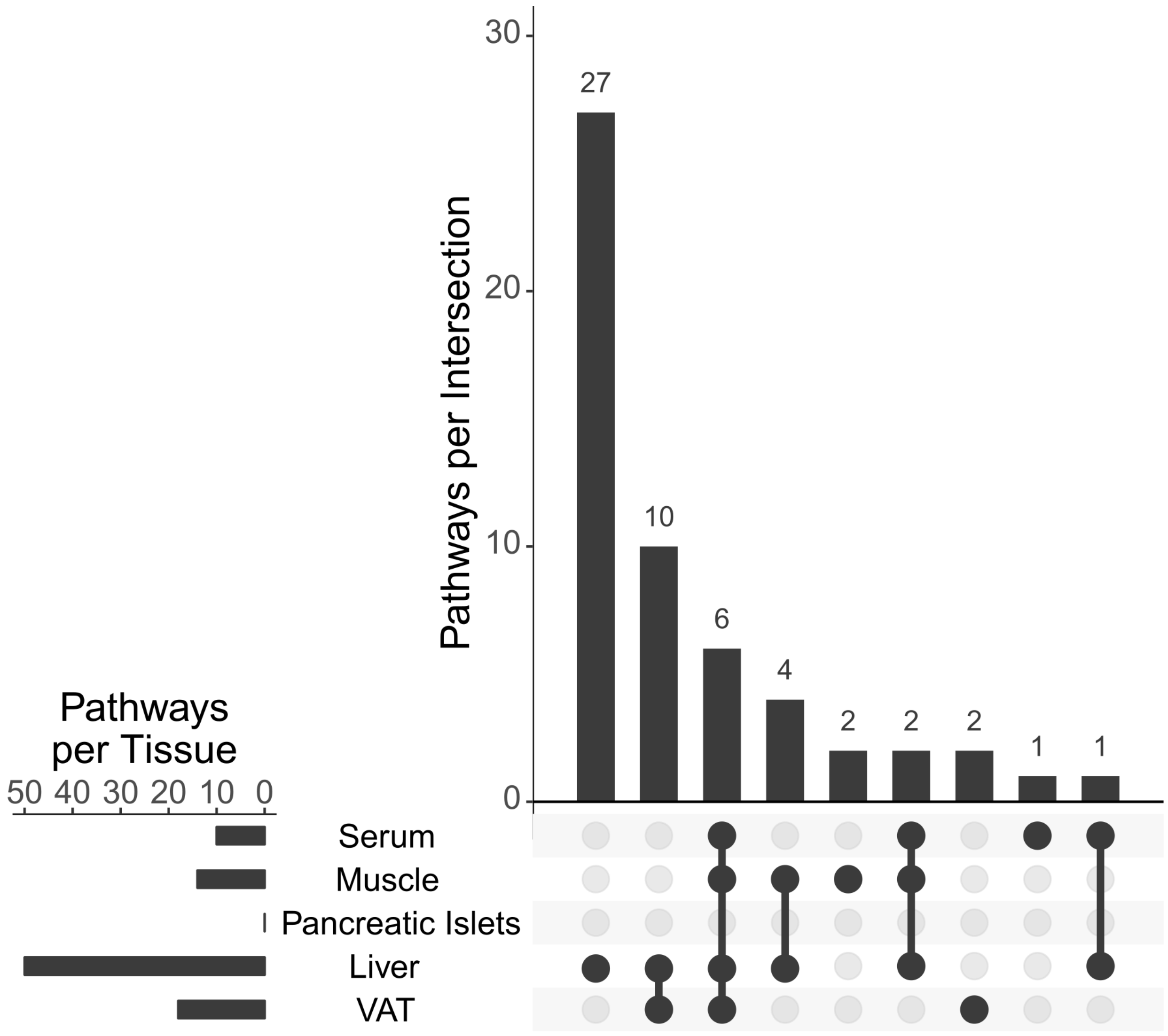

Supplementary Figure S17: Number of significantly enriched pathways per tissue. Pathways are computed from MBROLE 2.0[11] based on collections of significant metabolites for each tissue (Methods - Statistical analysis; Supplementary Note - Pathway and taxonomy enrichment analysis). The dot-matrix shows metabolic pathways unique and shared among tissues. The bottom-left barplot shows the number of statistically enriched pathways per tissue. The top barplot shows the number of shared pathways per intersection/combination.

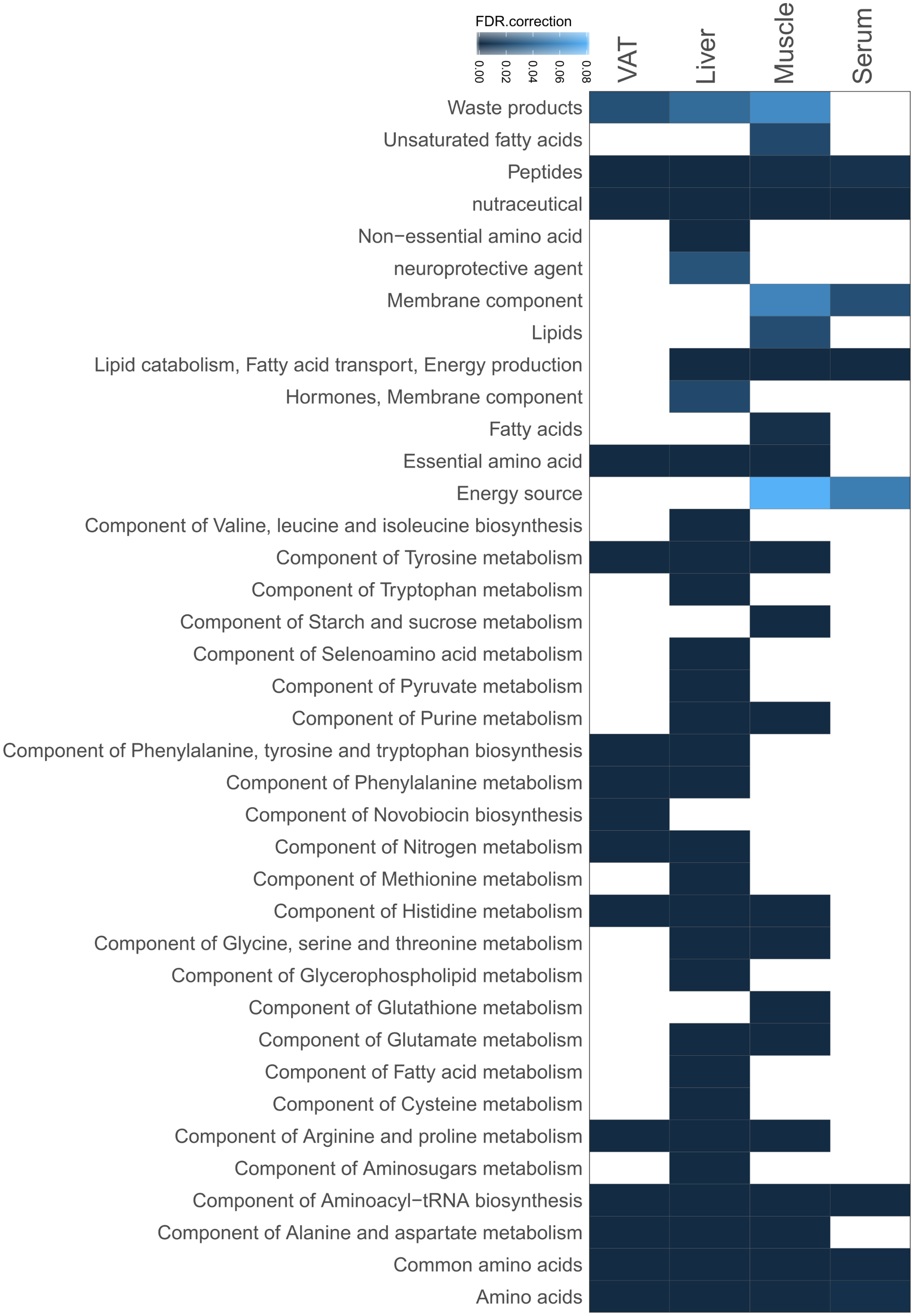
FDR.correction
0.00
0.02
0.04
0.06
0.08
VAT
Liver
Muscle
Serum
Waste products
Unsaturated fatty acids
Peptides
nutraceutical
Non−essential amino acid
neuroprotective agent
Membrane component
Lipids
Lipid catabolism, Fatty acid transport, Energy production
Hormones, Membrane component
Fatty acids
Essential amino acid
Energy source
Component of Valine, leucine and isoleucine biosynthesis
Component of Tyrosine metabolism
Component of Tryptophan metabolism
Component of Starch and sucrose metabolism
Component of Selenoamino acid metabolism
Component of Pyruvate metabolism
Component of Purine metabolism
Component of Phenylalanine, tyrosine and tryptophan biosynthesis
Component of Phenylalanine metabolism
Component of Novobiocin biosynthesis
Component of Nitrogen metabolism
Component of Methionine metabolism
Component of Histidine metabolism
Component of Glycine, serine and threonine metabolism
Component of Glycerophospholipid metabolism
Component of Glutathione metabolism
Component of Glutamate metabolism
Component of Fatty acid metabolism
Component of Cysteine metabolism
Component of Arginine and proline metabolism
Component of Aminosugars metabolism
Component of Aminoacyl−tRNA biosynthesis
Component of Alanine and aspartate metabolism
Common amino acids
Amino acids

Supplementary Figure S18: Metabolic biological roles enriched in various tissues. Biological roles enrichment was obtained from MBROLE2.0[11] for significant metabolites in the corresponding tissue (Methods - Statistical analysis; Supplementary Note - Pathway and taxonomy enrichment analysis). Color-coding is based on FDR corrected p-values (Supplementary Table S5).

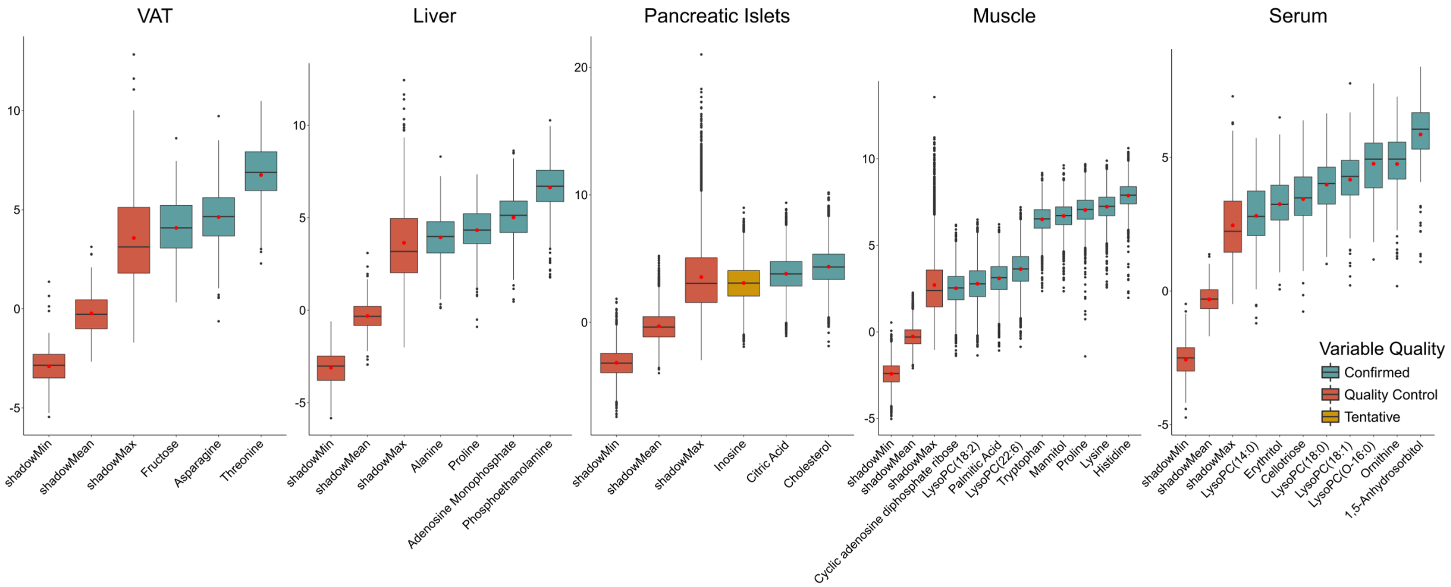

Supplementary Figure S19: All-relevant variables for each tissue that best discern between the decision classes selected by Boruta (Supplementary Note - Multivariate Statistical Analysis)[13]. The color-coding of the boxplots is explained in the legend. Confirmed variables are those that are statistically significant when compared to random synthetic variables generated by Boruta. The variables marked as tentative are those that are borderline significant, and those marked as quality control variables are those that Boruta generates randomly to assess the quality of each experimental variable. The red dot shows the mean value of permutations. The x axis shows the importance of the variable which is assessed by measuring the loss of the random forest classification accuracy when permuting the variable's values among objects.

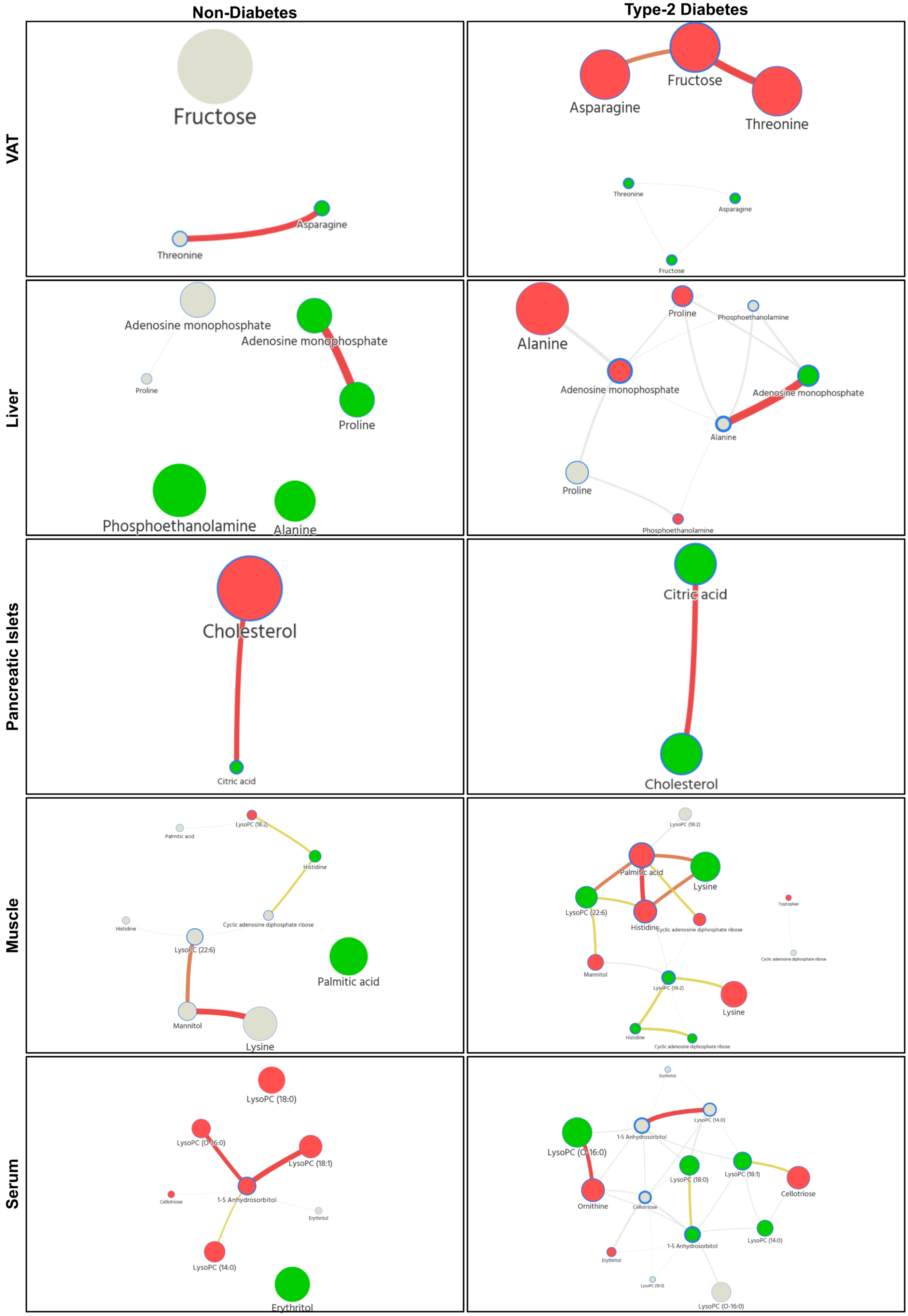
Non-Diabetes
Type-2 Diabetes
VAT
Liver
Pancreatic Islets
Muscle
Serum
Fructose
Asparagine
Threonine
Fructose
Asparagine
Threonine
Threonine
Asparagine
Fructose
Adenosine monophosphate
Adenosine monophosphate
Proline
Proline
Phosphoethanolamine
Alanine
Alanine
Proline
Phosphoethanolamine
Adenosine monophosphate
Adenosine monophosphate
Alanine
Proline
Phosphoethanolamine
Cholesterol
Citric acid
Citric acid
Cholesterol
LysoPC (18:2)
Palmitic acid
Histidine
Cyclic adenosine diphosphate ribose
Histidine
LysoPC (22:6)
Palmitic acid
Mannitol
Lysine
LysoPC (18:2)
Palmitic acid
Lysine
LysoPC (22:6)
Histidine
Cyclic adenosine diphosphate ribose
Tryptophan
Cyclic adenosine diphosphate ribose
Mannitol
LysoPC (18:2)
Lysine
Histidine
Cyclic adenosine diphosphate ribose
LysoPC (18:0)
LysoPC (O-16:0)
LysoPC (18:1)
Cellotriose
1-5 Anhydrosorbitol
Erythritol
LysoPC (14:0)
Erythritol
Erythritol
LysoPC (14:0)
1-5 Anhydrosorbitol
LysoPC (O-16:0)
LysoPC (18:0)
LysoPC (18:1)
Cellotriose
Ornithine
Cellotriose
1-5 Anhydrosorbitol
LysoPC (14:0)
Erythritol
LysoPC (18:0)
LysoPC (O-16:0)

Supplementary Figure S20: Visualization of rule-based classifers acquired from ROSETTA with VisuNet[14,17,18]. ROSETTA was ran as described in Supplementary Note - Rule-based Classification and rules were saved in a line-by-line format. Next VisuNet was employed to visualize the rule-models. Rows represent tissues. The left column visualizes the combinations of metabolites deciding for Non-Diabetes, while the right one for type 2 diabetes in the corresponding tissue. The size of the node shows the mean support value. The color of the node shows the level of the metabolite: Green: Low; Grey: Medium; Red: High. The border thickness of the node shows the number of rules in which the node participates at. The color of the edges implies statistical significance of the connections and it scales from grey to dark red.

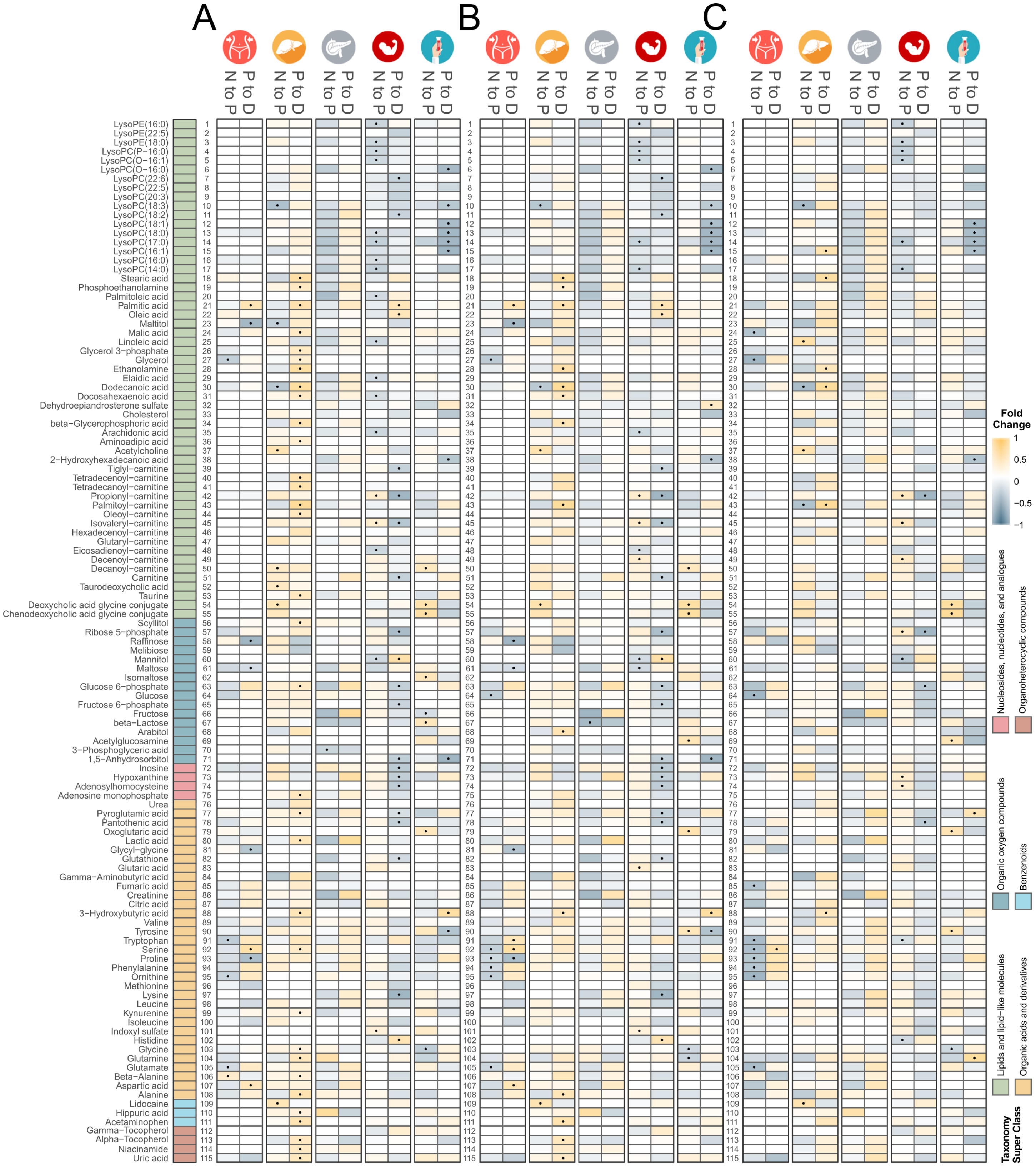

Supplementary Figure S21: Statistically significant metabolites showing the progression of type 2 diabetes. The leftmost column shows a manually curated classification of the metabolites based on HMDB's Super Class annotation[10]. Columns of the heatmaps show comparison between Controls and Pre-diabetes (N to P) and comparison between Pre-diabetes and type 2 diabetes (P to D). A black dot implies statistical significance (Mann-Whitney U test permuted p-value<0.1; Methods - Statistical analysis). The background color in the cells represents fold-change level and the color-coding is explained in

the legend. **A)** Metabolite intensities without covariate correction; **B)** Metabolite intensities corrected for BMI, Age, Gender and Sample Weight; **C)** Metabolite intensities corrected for BMI, Age, Gender, Sample Weight, HbA1c and GSIS.

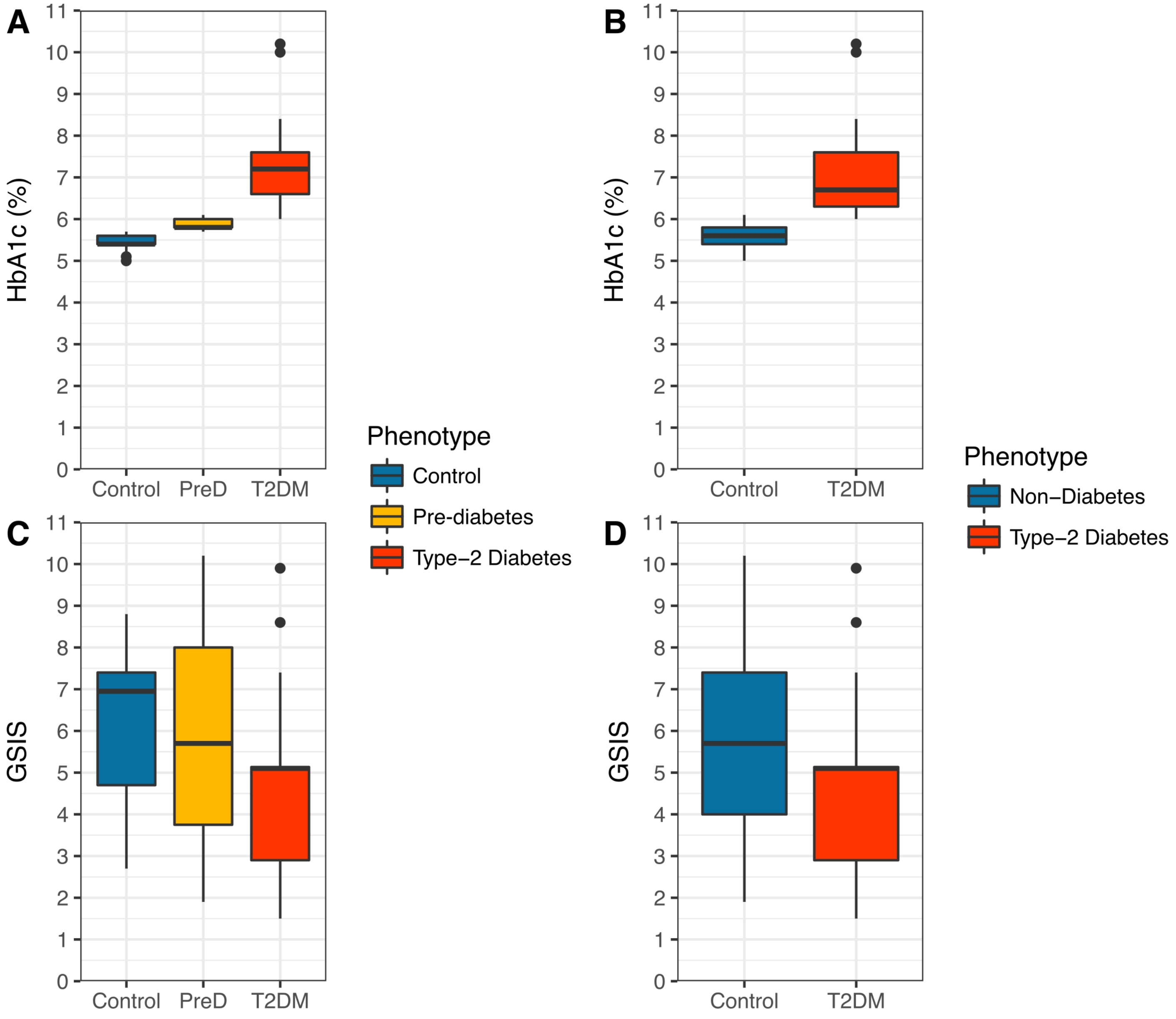

Supplementary Figure S22: Levels of HbA1c (% of mmol/mol) **(A and B)** and GSIS levels (mmol in liters of glucose) **(C and D)** for **A)** and **C)** three phenotypic classes (Controls, Pre-diabetes and Type-2 Diabetes), and **B)** and **D)** two phenotypic classes (Non-diabetes and Type-2 Diabetes).

REFERENCES

1. Suhre, K. *et al.* Metabolic Footprint of Diabetes: A Multiplatform Metabolomics Study in an Epidemiological Setting. *PLOS ONE* **5**, e13953 (2010).
2. Evans, A. M., DeHaven, C. D., Barrett, T., Mitchell, M. & Milgram, E. Integrated, Nontargeted Ultrahigh Performance Liquid Chromatography/Electrospray Ionization Tandem Mass Spectrometry Platform for the Identification and Relative Quantification of the Small-Molecule Complement of Biological Systems. *Anal. Chem.* **81**, 6656–6667 (2009).
3. Ohta, T. *et al.* Untargeted Metabolomic Profiling as an Evaluative Tool of Fenofibrate-Induced Toxicology in Fischer 344 Male Rats. *Toxicol. Pathol.* **37**, 521–535 (2009).
4. Suhre, K. *et al.* Human metabolic individuality in biomedical and pharmaceutical research. *Nature* **477**, 54–60 (2011).
5. A, J. *et al.* Extraction and GC/MS Analysis of the Human Blood Plasma Metabolome. *Anal. Chem.* **77**, 8086–8094 (2005).
6. Gullberg, J., Jonsson, P., Nordström, A., Sjöström, M. & Moritz, T. Design of experiments: an efficient strategy to identify factors influencing extraction and derivatization of Arabidopsis thaliana samples in metabolomic studies with gas chromatography/mass spectrometry. *Anal. Biochem.* **331**, 283–295 (2004).
7. Schauer, N. *et al.* GC–MS libraries for the rapid identification of metabolites in complex biological samples. *FEBS Lett.* **579**, 1332–1337 (2005).
8. Do, K. T., Rasp, D. J. N.-P., Kastenmüller, G., Suhre, K. & Krumsiek, J. MoDentify: phenotype-driven module identification in metabolomics networks at different resolutions. *Bioinformatics* doi:10.1093/bioinformatics/bty650
9. Krumsiek, J., Suhre, K., Illig, T., Adamski, J. & Theis, F. J. Gaussian graphical modeling reconstructs pathway reactions from high-throughput metabolomics data. *BMC Syst. Biol.* **5**, 21 (2011).
10. Wishart, D. S. *et al.* HMDB 4.0: the human metabolome database for 2018. *Nucleic Acids Res.* **46**, D608–D617 (2018).
11. López-Ibáñez, J., Pazos, F. & Chagoyen, M. MBROLE 2.0—functional enrichment of chemical compounds. *Nucleic Acids Res.* **44**, W201–W204 (2016).
12. Ganna, A. *et al.* Large-scale non-targeted metabolomic profiling in three human population-based studies. *Metabolomics* **12**, 4 (2015).
13. Kursa, M. B. & Rudnicki, W. R. Feature Selection with the Boruta Package. *J. Stat. Softw.* **36**, 1–13 (2010).
14. Øhrn, A. & Komorowski, J. ROSETTA – A Rough Set Toolkit for Analysis of Data. in *Proc. Third International Joint Conference on Information Sciences* 403–407 (1997).
15. Pawlak, Z. Rough sets. *Int. J. Comput. Inf. Sci.* **11**, 341–356 (1982).
16. Quinlan, J. R. Induction of decision trees. *Mach. Learn.* **1**, 81–106 (1986).
17. Garbulowski, M. *R.ROSETTA. Contribute to mategarb/R.ROSETTA development by creating an account on GitHub*. (2018).
18. Smolinska, K., Garbulowski, M., Komorowski, J. & Diamanti, K. VisuNet: Visualizing Networks of interacting features in rule-based classifiers. *Prep.* (2018).
